# Cortical astrocytes independently regulate sleep depth and duration via separate GPCR pathways

**DOI:** 10.7554/eLife.63329

**Published:** 2021-03-17

**Authors:** Trisha V Vaidyanathan, Max Collard, Sae Yokoyama, Michael E Reitman, Kira E Poskanzer

**Affiliations:** 1 Neuroscience Graduate Program, University of California, San Francisco San Francisco United States; 2 Department of Biochemistry & Biophysics, University of California, San Francisco San Francisco United States; 3 Kavli Institute for Fundamental Neuroscience San Francisco United States; Boston Children's Hospital United States; University of Texas at Austin United States

**Keywords:** sleep, astrocytes, slow-wave activity, cortex, two-photon imaging, chemogenetics, Mouse

## Abstract

Non-rapid eye movement (NREM) sleep, characterized by slow-wave electrophysiological activity, underlies several critical functions, including learning and memory. However, NREM sleep is heterogeneous, varying in duration, depth, and spatially across the cortex. While these NREM sleep features are thought to be largely independently regulated, there is also evidence that they are mechanistically coupled. To investigate how cortical NREM sleep features are controlled, we examined the astrocytic network, comprising a cortex-wide syncytium that influences population-level neuronal activity. We quantified endogenous astrocyte activity in mice over natural sleep and wake, then manipulated specific astrocytic G-protein-coupled receptor (GPCR) signaling pathways in vivo. We find that astrocytic Gi- and Gq-coupled GPCR signaling separately control NREM sleep depth and duration, respectively, and that astrocytic signaling causes differential changes in local and remote cortex. These data support a model in which the cortical astrocyte network serves as a hub for regulating distinct NREM sleep features.

## eLife digest

Sleep has many roles, from strengthening new memories to regulating mood and appetite. While we might instinctively think of sleep as a uniform state of reduced brain activity, the reality is more complex. First, over the course of the night, we cycle between a number of different sleep stages, which reflect different levels of sleep depth. Second, the amount of sleep depth is not necessarily even across the brain but can vary between regions.

These sleep stages consist of either rapid eye movement (REM) sleep or non-REM (NREM) sleep. REM sleep is when most dreaming occurs, whereas NREM sleep is particularly important for learning and memory and can vary in duration and depth. During NREM sleep, large groups of neurons synchronize their firing to create rhythmic waves of activity known as slow waves. The more synchronous the activity, the deeper the sleep.

Vaidyanathan et al. now show that brain cells called astrocytes help regulate NREM sleep. Astrocytes are not neurons but belong to a group of specialized cells called glia. They are the largest glia cell type in the brain and display an array of proteins on their surfaces called G-protein-coupled receptors (GPCRs). These enable them to sense sleep-wake signals from other parts of the brain and to generate their own signals. In fact, each astrocyte can communicate with thousands of neurons at once. They are therefore well-poised to coordinate brain activity during NREM sleep.

Using innovative tools, Vaidyanathan et al. visualized astrocyte activity in mice as the animals woke up or fell asleep. The results showed that astrocytes change their activity just before each sleep–wake transition. They also revealed that astrocytes control both the depth and duration of NREM sleep via two different types of GPCR signals. Increasing one of these signals (Gi-GPCR) made the mice sleep more deeply but did not change sleep duration. Decreasing the other (Gq-GPCR) made the mice sleep for longer but did not affect sleep depth.

Sleep problems affect many people at some point in their lives, and often co-exist with other conditions such as mental health disorders. Understanding how the brain regulates different features of sleep could help us develop better – and perhaps more specific – treatments for sleep disorders. The current study suggests that manipulating GPCRs on astrocytes might increase sleep depth, for example. But before work to test this idea can begin, we must first determine whether findings from sleeping mice also apply to people.

## Introduction

Sleep is characterized by distinct electrophysiological features that reflect the rhythmic activity of large populations of neurons. One phase of sleep—non-rapid eye movement (NREM) sleep—is critical for several important functions including memory consolidation/destabilization and synaptic homeostasis (Klinzing et al., 2019; Genzel et al., 2014; Kim et al., 2019; Tononi and Cirelli, 2014; Diekelmann and Born, 2010; Tononi and Cirelli, 2006; Tononi and Cirelli, 2020; Ji and Wilson, 2007). These functions are thought to require slow-wave activity (SWA), the distinct oscillatory pattern of neural activity in the cortex that occurs during NREM sleep and differentiates it from the relatively desynchronized activity during wakefulness and REM sleep. However, neural activity during NREM sleep is not uniform over the course of sleep, but varies in duration and depth (as measured by SWA intensity). Past work has demonstrated that NREM sleep duration and depth can be independently controlled (Dijk and Beersma, 1989; Patrick and Gilbert, 1896). Indeed, the circuit mechanisms known to underlie sleep depth and duration are largely independent from each other and operate on very different time-scales: sleep duration is mediated by subcortical nuclei that receive direct input from circadian centers and drive sleep/wake transitions through release of neuromodulatory signals (Holst and Landolt, 2018; Saper and Fuller, 2017; Lee and Dan, 2012). On the other hand, SWA intensity is largely regulated by cortical and thalamocortical circuits (Chen et al., 2012; Steriade et al., 1993; Lemieux et al., 2015; Volgushev et al., 2006; Steriade and Timofeev, 2003; Sheroziya and Timofeev, 2014; Amzica and Steriade, 1995; Sanchez-Vives and McCormick, 2000). While these two physiological measures of sleep have been mostly described in non-overlapping mechanistic terms, there is also physiological evidence that sleep depth and duration can be coupled. For example, cortical calcium (Ca^2+^) signaling can act on a millisecond time-scale to modulate cortical synchrony during SWA while also engaging longer term signaling cascades that regulate the sleep/wake cycle (Ode et al., 2017; Tatsuki et al., 2016). Thus, the extent to which the neural mechanisms underlying sleep depth and duration are linked remains unclear.

The cortex—where mammalian sleep is most often measured—is a brain region where neural mechanisms underlying sleep duration and sleep depth coincide: many neuromodulatory nuclei associated with sleep/wake transitions send direct projections to the cortex (Björklund and Lindvall, 1978; Woolf, 1991; Loughlin et al., 1986; Panula et al., 1989), and the cortex plays an instrumental role in generating and propagating SWA during sleep (Volgushev et al., 2006; Sanchez-Vives and McCormick, 2000; Niethard et al., 2018; Stroh et al., 2013; Luczak et al., 2007; Massimini et al., 2004; Krone, 2020; Sanchez-Vives and Mattia, 2014; Lemieux et al., 2014). Further, cortical SWA intensity can be locally regulated, leading to heterogeneity of SWA across cortex (Huber et al., 2004; Funk et al., 2016; Siclari and Tononi, 2017). However, how the cortex integrates separate regulatory signals to orchestrate activity across sleep and wake is unknown. In untangling sleep mechanisms, both in cortex and throughout the brain, the historical focus has almost exclusively been on neurons and neuronal circuits. Yet astrocytes—the largest class of non-neuronal brain cells—are also situated to play critical roles in sleep regulation within the cortex. Astrocytes tile the cortex, can participate in bidirectional communication with thousands of neurons (Halassa et al., 2007; Allen and Barres, 2005; Bushong et al., 2002; Bazargani and Attwell, 2016), exhibit morphological and transcriptional changes during sleep (Bellesi et al., 2015), and regulate SWA under anesthesia (Szabó et al., 2017; Poskanzer and Yuste, 2016; Durkee et al., 2019). Further, multiple canonical astrocytic functions are also associated with sleep/wake regulation, including regulation of extracellular glutamate (Poskanzer and Yuste, 2016; Poskanzer and Yuste, 2011), extracellular ion dynamics (Ding et al., 2016), release of neurotransmitters (Halassa et al., 2009; Papouin et al., 2017; Fellin et al., 2009), and metabolic regulation (Petit and Magistretti, 2016; Bellesi et al., 2018; DiNuzzo and Nedergaard, 2017).

Astrocyte physiology is primarily measured via intracellular Ca^2+^dynamics, which vary widely in size, shape, and location, and can propagate within or even between cells (Wang et al., 2019; Khakh and McCarthy, 2015; Shigetomi et al., 2013; Shigetomi et al., 2016; Guerra-Gomes et al., 2017). Because imaging complex astrocyte Ca^2+^ activity in vivo is relatively new, it remains unknown whether these diverse astrocytic Ca^2+^ dynamics map onto different circuit functions. However, the potential of astrocytes to influence large populations of cortical neurons across different time-scales is significant (Stobart et al., 2018; Lind et al., 2013). The majority of astrocyte Ca^2+^ activity is thought to result from upstream activation of G-protein coupled receptors (GPCRs) (Durkee et al., 2019; Di Castro et al., 2011; Agulhon et al., 2008; Kofuji and Araque, 2021). Importantly, many astrocytic GPCRs are activated by neuromodulators, including those associated with sleep/wake regulation, such as norepinephrine, acetylcholine, and histamine. Since GPCRs regulate a diverse array of Ca^2+^-dependent intracellular signals on many different time-scales (Grundmann and Kostenis, 2017; Kholodenko et al., 2010), they are prime candidates for differentially regulating individual features of NREM sleep, such as duration and depth. A downstream target of GPCRs, the inositol triphosphate type two receptor (IP_3_R2), has been recently shown to be involved in sleep regulation (Bojarskaite et al., 2020). In astrocytes, both Gi- and Gq-coupled GPCRs activate IP_3_R2s and lead to increases in intracellular Ca^2+ ^(Durkee et al., 2019; Mariotti et al., 2016; Nagai et al., 2019), while also engaging separate signaling cascades. Despite this, scant attention has been paid to whether the activation of different astrocytic GPCRs, and resulting Ca^2+^ signals, have differential effects on the surrounding neural circuit. Indeed, GPCR signaling in astrocytes may underlie mechanisms by which astrocytes perform multiple, parallel functions in the neural circuit.

Here, we leveraged a recently developed image analysis tool that captures the spatiotemporal complexity of astrocyte Ca^2+^ dynamics (Wang et al., 2019) and astrocyte-specific chemogenetics to investigate the mechanisms by which cortical astrocytes both link and independently regulate different features of NREM sleep via GPCR signaling. To do this, we carried out in vivo two-photon (2P) imaging of astrocyte Ca^2+^ while recording electrophysiological sleep rhythms to examine astrocyte Ca^2+^ changes across natural sleep and wake. We find that endogenous Ca^2+^ activity is inversely correlated with SWA and exhibits bidirectional changes prior to sleep-wake transitions. Using chemogenetics to selectively manipulate astrocytic Gi- and Gq-GPCR pathways, we demonstrate that astrocytes actively regulate both NREM sleep duration and depth, via separate GPCR signaling pathways: astrocytic Gi-induced Ca^2+^ is sufficient to increase SWA (sleep depth), while sleep-wake transitions (sleep duration) is dependent on Gq-GPCRs. We demonstrate a role for astrocytes in both local and cortex-wide sleep regulation; manipulating astrocytic Ca^2+^ in primary visual cortex (V1) alters not only local SWA, but also affects SWA in contralateral frontal cortex (FC). Further, we find that while local changes in SWA arise from greater changes in delta waves, remote SWA effects in FC are due to increases in slow oscillations. Since these two slow waves underlie different functions, our data support the concept that astrocytes exert different effects on neuronal populations depending on both the type of GPCR activated *and* their localization within cortical circuits. Together, our data support a role for the cortical astrocytic network as a hub for the regulation of sleep depth and duration across cortex.

## Results

### Accurate detection of astrocyte Ca^2+^ events in vivo across sleep and wake

**Figure 1.**
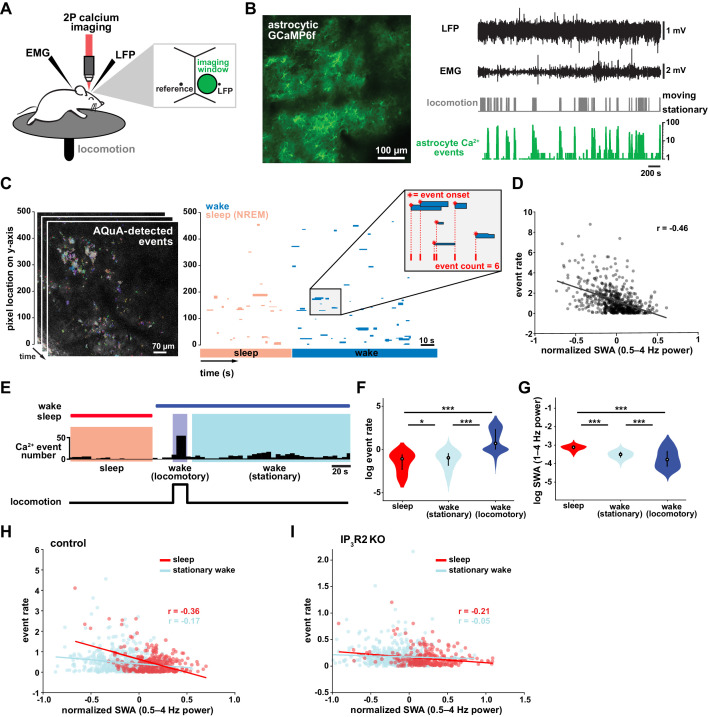
Cortical astrocyte Ca^2+^ event rate and SWA are negatively correlated across behavioral states. (**A**) Experimental in vivo imaging and electrophysiology setup. Mice expressing astrocytic GCaMP6f were head-fixed on a horizontal treadmill to record astrocyte Ca^2+^, LFP, EMG, and locomotion. (**B**) Left: example image of GCaMP6F expression in L2/3 V1 astrocytes in an awake, head-fixed mouse. Right: example of LFP, EMG, locomotion, and astrocyte Ca^2+^ event data using this experimental setup. (**C**) Left: AQuA (Wang et al., 2019) detects astrocyte Ca^2+^ events in 3 min GCaMP time series. Right: representative spatiotemporal plot of AQuA-identified Ca^2+^ events displaying event time and duration on the x-axis, and the unidimensional (**y**) spatial extent on the y-axis. Events are color-coded by behavioral state (wake=blue, NREM sleep=pink). Inset demonstrates that event count throughout paper is quantified using event onset. (**D**) Ca^2+^ event rate and SWA are negatively correlated (Pearson’s correlation, p<0.0005). Each point represents a single 2 min bin (for all data in this figure except panel I, n = 4 mice, 19 hr). (**E**) Example of Ca^2+^ event rate (bottom row) across three behavioral states: NREM sleep, locomotory wake, and stationary wake. (**F**) Across animals, Ca^2+^ event rate is lowest during sleep, higher during stationary wake, and highest during locomotory wake, while (**G**) the inverse is true for SWA (for F and G, rank sum test, data are represented as median, 25th and 75th percentile). (**H**) Ca^2+^ event rate and SWA are negatively correlated within each behavioral period: sleep (red, Pearson’s correlation, p<0.0005) and stationary wake (cyan, Pearson’s correlation, p<0.0005) (**I**), but Ca^2+^ events in IP_3_R2 KO mice are less correlated with SWA in sleep (Pearson’s correlation, p<0.0005) and stationary wake (Pearson’s correlation, p>0.05, n = 5 mice, 22 hr).

To study the role of astrocytes in sleep regulation, we conducted 2P imaging of astrocyte Ca^2+^ dynamics as animals naturally transitioned between sleep and wake states (Niethard et al., 2018; Seibt et al., 2017). To specifically express the Ca^2+^ indicator GCaMP6f in cortical astrocytes, we injected mice with *AAV-GFAP-GCaMP6f* 2–4 weeks before experiments (Figure 1B, left). Electrodes were implanted for local field potential (LFP) and electromyogram (EMG) recordings (Figure 1B, right) to assess sleep state. During recording sessions, mice were head-fixed on a horizontal treadmill, and locomotion was recorded (Figure 1A). To control for the effect of circadian rhythm and sleep pressure, all recording sessions took place between ZT 2–5. Experiments were conducted after mice had been previously habituated to head-fixation to allow natural sleep. To analyze astrocyte Ca^2+^ activity, we used our recent tool, AQuA (Wang et al., 2019), an event-based approach to detect spatiotemporally distinct Ca^2+^ events without predetermined regions-of-interest (ROIs). This allowed automatic detection of individual astrocyte Ca^2+^ events, independent of size and shape, across sleep and wake (Figure 1C, Video 1).

**Figure 1—figure supplement 1.**
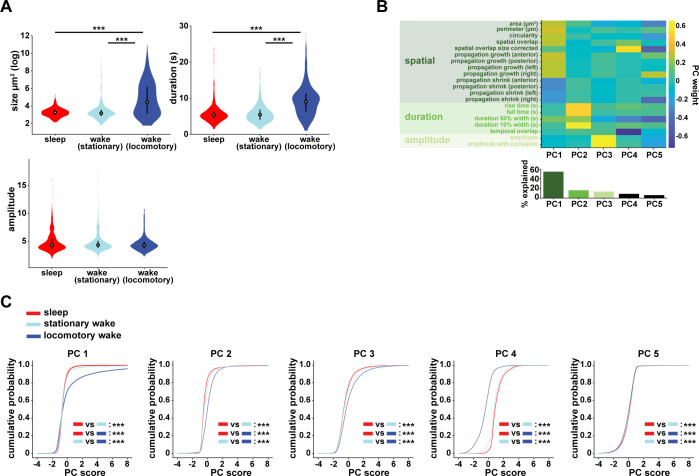
PCA reveals that behavioral states can be characterized by unique properties of astrocyte Ca^2+^ events. (**A**) Individual quantification of Ca^2+^ event size (top left), duration (top right), and amplitude (bottom) across sleep, stationary wake, and locomotory wake reveal significant differences in size and duration between locomotory wake and the other two states, but no difference between sleep and stationary wake (rank sum test). Data are represented as median, 25th and 75th percentile (n = 4 mice, 19 hr, 1089 locomotory wake periods, 443 stationary wake periods, 285 sleep periods). (**B**) Top: Weight matrix extracted from PCA, incorporating 20 Ca^2+^ event properties obtained with AQuA (n = 4 mice, 19 hr, 87,189 events). PC 1 largely represents spatial features, PC 2 represents temporal features, and PC 3 represents features associated with amplitude. Bottom: Percent of variance explained by each PC. (**C**) Cumulative distribution of the score for each PC for sleep, stationary wake, and locomotory wake events demonstrates significant differences among all three states (two-sample Kolmogorov-Smirnov test).

**Video 1.** Endogenous cortical astrocytic Ca^2+^ activity. Example video of 20 min of endogenous in vivo GCaMP activity in layer 2/3 V1 cortical astrocytes, with AQuA-detected events overlaid. Behavioral state is annotated in the upper left corner. Frame rate = 30 Hz. Scale bar = 50 μm.

### Cortical astrocyte Ca^2+^ frequency and SWA are negatively correlated across behavioral states

To investigate the relationship between in vivo cortical astrocyte activity and NREM sleep, we first quantified the relationship between Ca^2+^ event rate and SWA (0.5–4 Hz power), a marker of NREM sleep depth. By dividing entire 2–3 hr recordings into two-min bins, we found that Ca^2+^ event rate and SWA were negatively correlated (Figure 1D), that is when SWA is low, astrocyte Ca^2+^ event rate is high, and vice versa. This finding suggests astrocytes may play roles regulating SWA, an idea supported by previous studies demonstrating that astrocyte Ca^2+^ plays a causal role in driving low frequency-dominated cortical states under anesthesia (Szabó et al., 2017; Poskanzer and Yuste, 2016; Fellin et al., 2009).

To determine whether the negative correlation between astrocyte Ca^2+^ and SWA is specific to a particular behavioral state, we analyzed our data by dividing recording periods into sleep, locomotory wake, and stationary wake (Figure 1E). We separated wake by locomotion to quantify Ca^2+^ dynamics independently from large Ca^2+^ bursts that occur with locomotion onset (Wang et al., 2019; Paukert et al., 2014; Nimmerjahn et al., 2009). As predicted by the negative correlation between event rate and SWA (Figure 1D), we found that Ca^2+^ event rate was highest during locomotory wake, lower during stationary wake, and lowest during sleep (Figure 1F). To confirm, we compared SWA in the three behavioral states and found an inverse relationship of Ca^2+^ event rate, namely SWA was highest during sleep, lower during stationary wake, and lowest during locomotory wake (Figure 1G). These findings are supported by recent work demonstrating the same pattern of Ca^2+^ activity across similar behavioral states, using an ROI-based image analysis approach (Bojarskaite et al., 2020), confirming that our event-based image analysis can generate comparable results when the same metrics are quantified. Together, these data demonstrate that changes in Ca^2+^ event frequency co-occur with major changes in behavioral state, consistent with levels of SWA.

To explore whether each behavioral state can be characterized by the types of astrocytic Ca^2+^ events that occur during these states, we first compared the events' size, duration, and amplitude. As predicted by the large, synchronous bursts observed during locomotion, we found locomotory wake Ca^2+^ events were larger in size and duration than events observed in the other two states. However, when we controlled for locomotion we did not find differences in size, duration, or amplitude of events between sleep and stationary wake when these features were compared individually (Figure 1—figure supplement 1A). However, astrocyte events have many other features beyond size, duration, and amplitude, such as event perimeter or propagation. Because of this spatiotemporal complexity, we next used a dimensionality reduction approach, implementing principal component analysis to explore whether astrocyte Ca^2+^ events differed among behavioral states. This approach allowed us to incorporate 20 different event features calculated by AQuA. We found that the first three principal components (PCs) represented spatial-, temporal-, and amplitude-related features respectively. We then focused on the five PCs that explained the most variance in the imaging data (Figure 1—figure supplement 1B) and compared them among the three behavioral states. While the largest differences in each PC were between locomotory wake and the other two states, we also found significant differences between sleep and stationary wake in all five PCs examined (Figure 1—figure supplement 1C). Together, this analysis demonstrates that while no state-specific differences are observed by comparisons of individual event features, there are unique spatial, temporal, and amplitude signatures of sleep-specific astrocyte Ca^2+^ events when multiple features are incorporated.

We next examined the relationship between astrocyte Ca^2+^ event frequency and SWA within stationary behavioral states and found, similar to Figure 1D, a negative correlation between Ca^2+^ frequency and SWA (Figure 1H). The strong association found between Ca^2+^ frequency and SWA during sleep, namely high Ca^2+^ activity during sleep periods of low SWA and vice versa, is suggestive of a possible role of astrocytic Ca^2+^ specifically in sleep depth. Lastly, we explored the role of IP_3_R2 in the relationship between astrocyte Ca^2+^ activity and SWA since IP_3_R2s are enriched in astrocytes (Zhang et al., 2014), underlie a significant fraction of astrocytic Ca^2+^ dynamics through Ca^2+^ release from intracellular stores (Beck et al., 2004), and IP_3_R2 KO mice show a total decrease in SWA during NREM sleep (Bojarskaite et al., 2020). To test whether the inverse relationship of astrocyte Ca^2+^ and SWA is dependent on IP_3_R2s, we imaged astrocyte Ca^2+^ dynamics over natural sleep and wake in IP_3_R2 KO mice (Petravicz et al., 2008). Similar to previous work (Srinivasan et al., 2015), we noted a reduction, but not complete abolishment, of Ca^2+^ events in IP_3_R2 KO mice. In IP_3_R2 KO mice, Ca^2+^ event rate and SWA were negatively correlated, but the correlation was decreased compared to controls (Figure 1I), suggesting the astrocyte-SWA relationship is at least partially dependent on the IP_3_R2. The change in correlation between control and IP_3_R2 KO was most dramatic in sleep, implicating IP_3_R2-dependent astrocytic Ca^2+^ signaling in the regulation of SWA intensity in the sleep state. Since both Gq- and Gi-GPCR signaling can increase Ca^2+^ in astrocytes through IP_3_R2s (Durkee et al., 2019; Nagai et al., 2019) and astrocytes express many GPCRs that have been implicated in sleep-wake regulation (Durkee et al., 2019; Di Castro et al., 2011; Agulhon et al., 2008; Kofuji and Araque, 2021), the relationship between astrocytic Ca^2+^ and SWA may result from astrocytic sensing of sleep-wake cues through GPCR signaling.

### Transitions from low to high SWA are centered around astrocyte Ca^2+^ events

**Figure 2.**
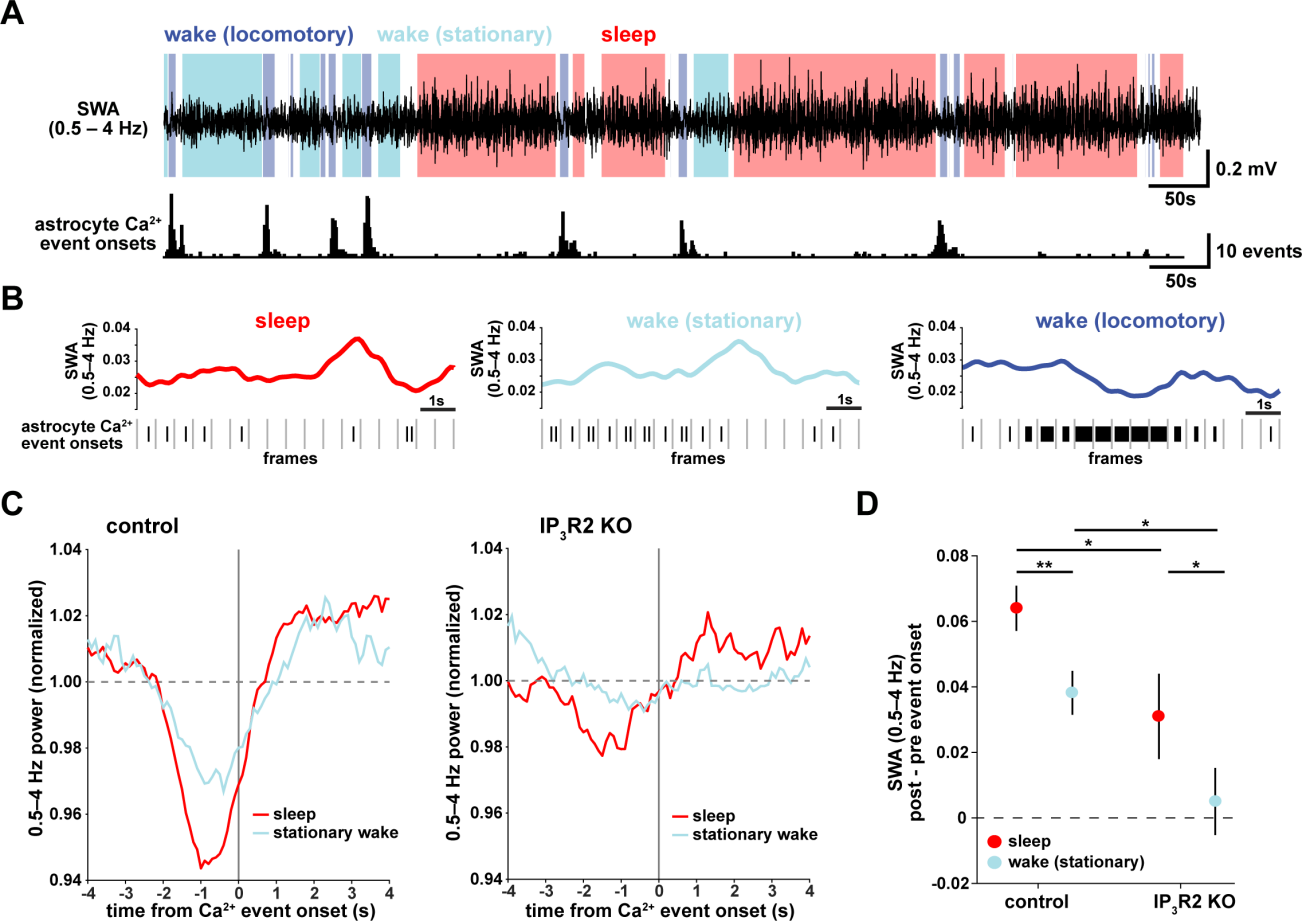
Astrocyte Ca^2+^ events characterize transitions from low to high SWA. (**A**) Example of SWA obtained by filtering LFP (0.5–4 Hz) recordings (top), with corresponding astrocyte Ca^2+^ events (bottom). Behavioral state denoted by color: sleep (red) and wake (locomotory in blue, stationary in cyan). (**B**) Example traces of SWA fluctuations for each behavioral state (top) and raster plot of Ca^2+^ events (below) to demonstrate that across behavioral states, SWA fluctuates at similar levels. (**C**) Left: Average Ca^2+^ event-triggered traces reveal astrocyte Ca^2+^ event onsets occur after a relative decrease in SWA and are followed by an increase in SWA during sleep (red) and stationary wake (cyan). Right: This relationship is diminished in IP_3_R2 KO mice, where less modulation around Ca^2+^ events is observed. Line width=SEM (control: n = 4 mice, 19 hr; IP_3_R2 KO: n = 5 mice, 22 hr). (**D**) SWA modulation across the 2 s before and after Ca^2+^ onset demonstrates modulation is highest during sleep (red) and dependent on expression of IP_3_R2 (rank sum test, data represented as mean± SEM).

To understand how astrocyte Ca^2+^ activity is related to SWA on a shorter time-scale, we asked whether consistent electrophysiological changes occur in the seconds around the onset of astrocyte Ca^2+^ events. As earlier, we separated the recordings by sleep, stationary wake, and locomotory wake states (Figure 2A). Although SWA was, by definition, highest during sleep, we also observed significant fluctuation between periods of relative high and low SWA within each behavioral state (Figure 2B). We next calculated Ca^2+^ event-triggered averages of SWA, separated by behavioral state. Because the majority of locomotory wake Ca^2+^ events were in bursts tied to locomotion onset, we focused on sleep and stationary wake states. We found a pattern in which Ca^2+^ events were preceded by decreases in SWA and followed by increases in SWA (Figure 2C, left). This modulation was significantly higher during sleep compared to stationary wake (Figure 2D). Further, this SWA modulation was decreased in IP_3_R2 KO mice (Figure 2C, right, Figure 2D), indicating partial dependence of this relationship on IP_3_R2s (as in Figure 1I).

This specific pattern of SWA change centered on astrocyte Ca^2+^ events—low SWA before astrocyte events and higher afterward—suggests an active role of astrocytes in regulating sleep depth. Specifically, we speculate that astrocytes may be associated with a homeostatic process that increases SWA in response to a transient decrease in SWA. Although we cannot determine this from the data shown here, several lines of evidence support this hypothesis: astrocytes exhibit Ca^2+^ increases in response to many neuromodulators associated with decreased low-frequency power (Ding et al., 2013; Khan et al., 2001; Takata et al., 2011; Shelton and McCarthy, 2000) and cortical astrocytes have the ability to increase low-frequency power (Szabó et al., 2017; Poskanzer and Yuste, 2016). If, in fact, astrocyte Ca^2+^ events are ‘triggered’ by decreases in SWA, we would expect to observe more Ca^2+^ events when SWA is low, which we indeed found in the correlation analysis above (Figure 1D,H). While many other cell types may also play roles in a SWA homeostatic process, we wondered whether astrocytes may be involved in the consistent increase in SWA that we observe after astrocyte Ca^2+^ event onsets (Figure 2C). To address this question, we next used chemogenetics to specifically manipulate GPCR pathways that shape astrocyte Ca^2+^ dynamics.

### Gi-driven astrocyte Ca^2+^ increases are sufficient to increase SWA during sleep

**Figure 3.**
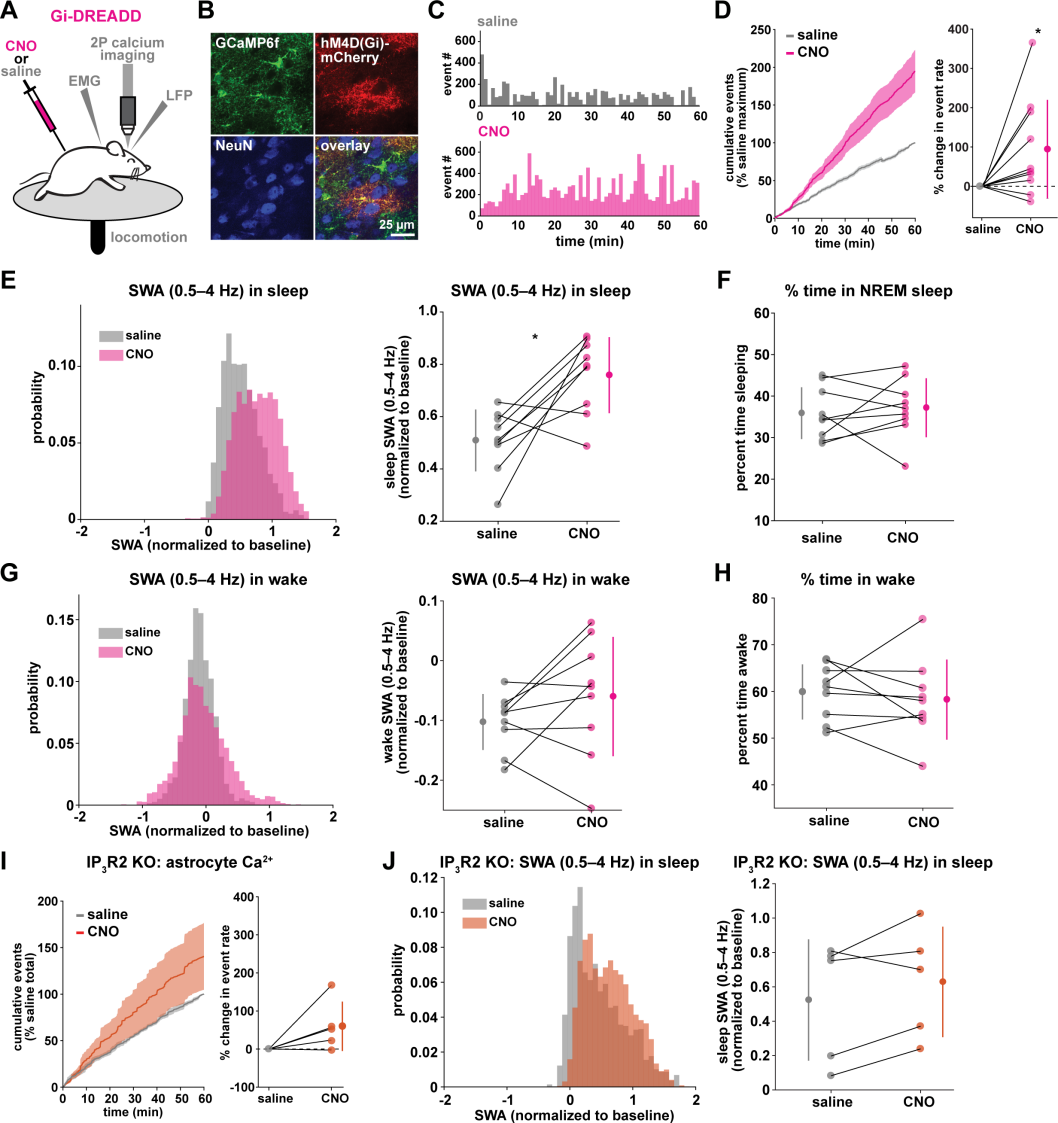
Astrocytic Gi-DREADD-driven Ca^2+^ increases are sufficient to increase SWA during sleep. (**A**) Experimental setup. Mice were co-injected with *GFAP-cyto-GCaMP6f* and *GFAP-hM4D(Gi)-mCherry* AAVs. After I.P. injection of either 1 mg/kg CNO or saline, astrocyte Ca^2+^, LFP, EMG, and locomotion were recorded. (**B**) Post-experiment immunohistochemistry demonstrates astrocyte-specific expression of Gi-DREADD and GCaMP6f. mCherry^+^ cells (red) exhibit typical astrocyte morphology and do not co-localize with neurons (NeuN, blue). (**C**) Representative astrocyte Ca^2+^ response in one animal in which CNO (pink, bottom) causes increased Ca^2+^ events compared with saline (gray, top). (**D**) Left: Cumulative Ca^2+^ event count for all mice after CNO (pink) shows higher event rate compared to saline (gray). Error bars=SEM. (n = 10 mice, 1 hr recordings). Right: Change in event rate for each mouse with CNO compared to saline (paired *t*-test). For panels D–J, data are represented by the mean for each individual animal, and the population as mean± SD. (**E**) Left: Administration of CNO (pink) results in rightward shift of the SWA distribution during sleep compared to saline (gray), using 5 s bins. Right: Summary statistics, by animal, show increased SWA during sleep after CNO. (for panels E–H, n = 9 mice, 2 hr recordings, paired *t*-test) (**F**) Percent time in NREM sleep does not differ between saline and CNO conditions. (**G**) Distribution of SWA (left) and summary statistics across mice (right) show that SWA during wake, in contrast with sleep (**E**), is unchanged between conditions. (**H**) Percent time awake, similar to time in NREM sleep (**F**), is similar between conditions. (**I**) CNO administration (orange) in IP_3_R2 KO mice expressing astrocytic Gi-DREADDs causes no significant change in Ca^2+^ event number compared to saline (gray) as shown by the cumulative event count (left, error bars = SEM) and summary statistics per mouse (right, paired *t*-test) (for experiments in I–J, n = 5 mice, 2 hr recordings). (**J**) Distribution of SWA during sleep (left) and summary statistics (right) show that, in contrast to controls (**E**), sleep SWA is unchanged between saline (gray) and CNO (orange) conditions in IP_3_R2 KO mice (paired *t*-test).

To test whether astrocyte Ca^2+^ may play a causal role in SWA control, we acutely manipulated cortical astrocyte Ca^2+^, since genetic manipulations—such as IP_3_R2 KO—can lead to compensatory developmental effects. Because IP_3_R2 can mediate the astrocyte-SWA relationship (Figures 1I and 2C–D), and both Gi- and Gq-GPCR mediated Ca^2+^ changes in astrocytes are dependent on the IP_3_R2 pathway (Durkee et al., 2019; Mariotti et al., 2016; Nagai et al., 2019), we chose to use Designer Receptors Exclusively Activated by Designer Drugs (DREADDs) (Roth, 2016) to selectively manipulate GPCR pathways in astrocytes. The inhibitory neurotransmitter GABA has been implicated in cortical synchrony during sleep through the mediation of synchronous DOWN states (Lemieux et al., 2015; Sheroziya and Timofeev, 2014; Zucca et al., 2017) and the excitatory neurotransmitter glutamate has been implicated in cortical UP states (Sanchez-Vives and McCormick, 2000; Poskanzer and Yuste, 2011). Astrocytes respond to both GABA and glutamate via Gi-GPCRs (via GABA_B_ and mGluR3 receptors in adults) (Durkee et al., 2019; Mariotti et al., 2016; Nagai et al., 2019). Thus, we chose the inhibitory human M4 muscarinic receptor DREADD (hM4Di) to selectively drive this well described Gi-GPCR pathway in astrocytes (Figure 3).

**Figure 3—figure supplement 1.**
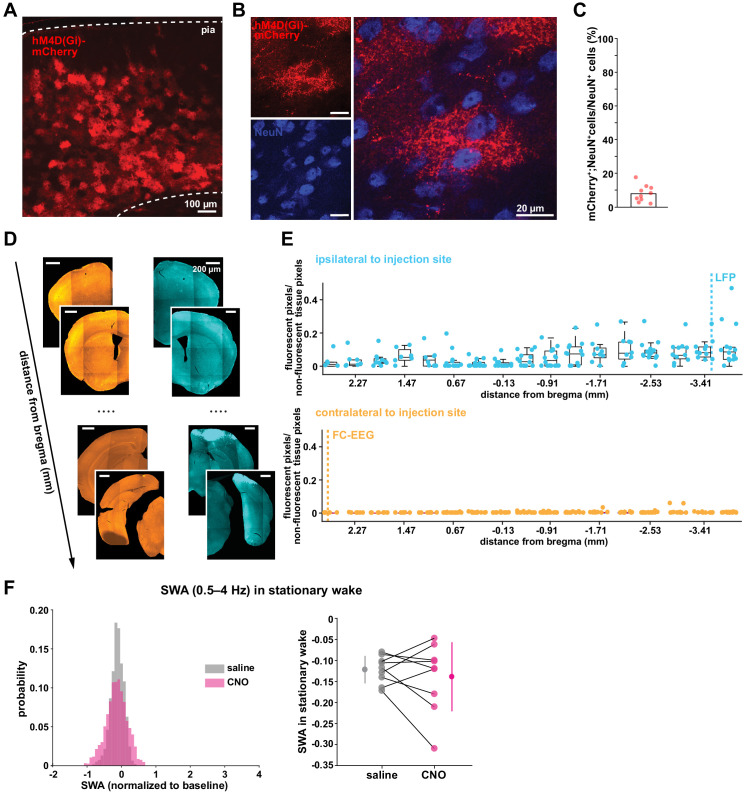
Astrocyte-specific expression of Gi-DREADDs across ipsilateral and contralateral cortex. (**A**) Representative post-experiment immunohistochemistry demonstrating widespread astrocyte-specific expression of Gi-DREADDs across cortical layers. Note stereotypical bush-like astrocytic morphology of mCherry^+^ cells. (**B**) Representative ×63 magnification confocal immunohistochemistry images of mCherry (red) and NeuN (blue) staining used to identify non-specific expression of DREADDs. (**C**) Percentage of NeuN^+^ identified cells that were also mCherry^+^. (**D**) Schematic demonstrating analysis of cortex-wide Gi-DREADD expression spread on the hemisphere ipsilateral and contralateral to the viral injection site. (**E**) Expression spread in brain slices sampled from rostral to caudal for the ipsilateral (top, cyan) and contralateral hemisphere (bottom, orange) demonstrates expression is highest in the ipsilateral rostral brain areas, near the LFP recording site (dashed line, cyan). There is no expression near the FC-EEG recording site (dashed line, yellow). Expression spread for each slice was calculated by normalizing the number of fluorescent pixels to the size of the brain tissue. Data represented with boxplots marking the medians, 25th and 75th percentiles. (**F**) Distribution of SWA (left), and summary statistics across mice (right, paired *t*-test) show that SWA during stationary wake, similar to all wake (Figure 3G) and in contrast with sleep (Figure 3E), is unchanged between saline and CNO conditions (n = 9 mice, 2 hr recordings). Data represented by mean for each animal and population mean± SD.

**Figure 3—figure supplement 2.**
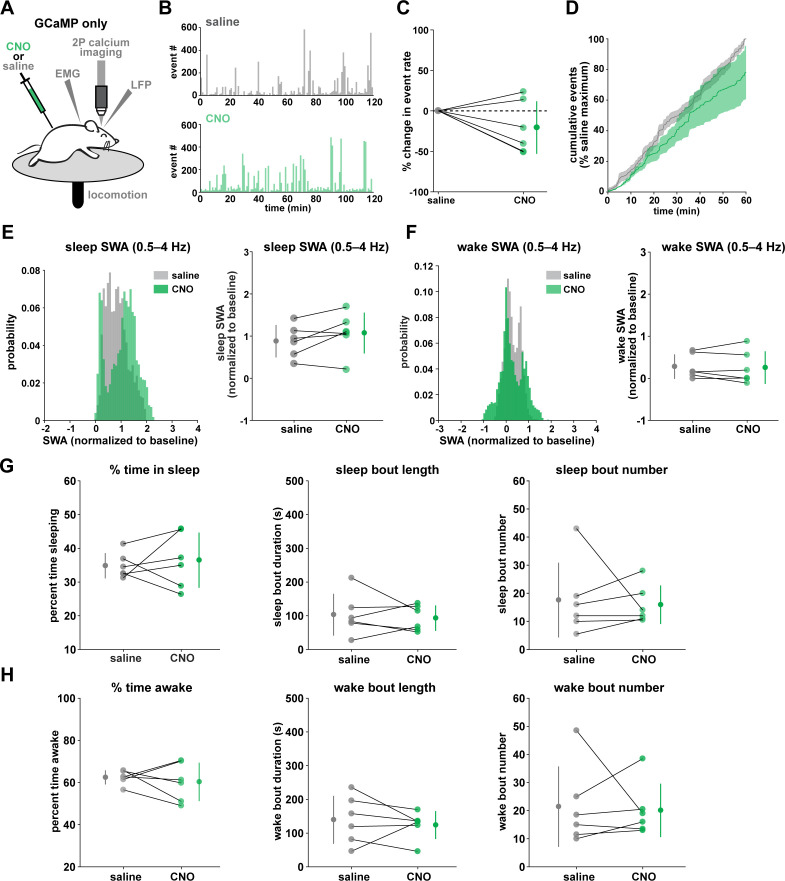
Systemic administration of CNO does not affect astrocyte Ca^2+^ dynamics, SWA, or sleep and wake behavior. (**A**) Experimental setup. Mice were injected with only *GFAP-cyto-GCaMP6f* AAV. After I.P. injection of either 1 mg/kg CNO or saline, astrocyte Ca^2+^, LFP, EMG, and locomotion were recorded to assess effects of CNO. (**B**) Example astrocyte Ca^2+^ response in one animal in which CNO (green, bottom) does not change Ca^2+^ event number compared with saline (gray, top). (**C**) Ca^2+^ event rate with CNO demonstrates no significant change across mice compared to saline (paired *t*-test, data represent mean for each mouse and population mean± SD, n = 6 mice, 2 hr recordings) (**D**) Cumulative Ca^2+^ event count for all mice shows no change between CNO (green) and saline (gray) conditions (error bars=SEM, n = 6 mice, 1 hr recordings). (**E**) Left: Administration of CNO (green) does not change SWA distribution during sleep compared to saline (gray), using 5 s bins. Right: Summary statistics show no change in SWA during sleep after CNO (for E–H, data represented by mean for each animal and population mean± SD, n = 6 mice, 2 hr recordings, paired *t*-test). (**F**) Left: CNO (green) does not change SWA distribution during wake compared to saline (gray), using 5 s bins. Right: No change in SWA during wake after CNO. (**G**) Time spent in NREM sleep (left), mean sleep bout length (middle), and mean sleep bout number (right) do not differ between saline and CNO conditions. (**H**) Time spent in wake (left), mean wake bout length (middle), and mean wake bout number (right) do not differ between saline and CNO conditions.

**Figure 3—figure supplement 3.**
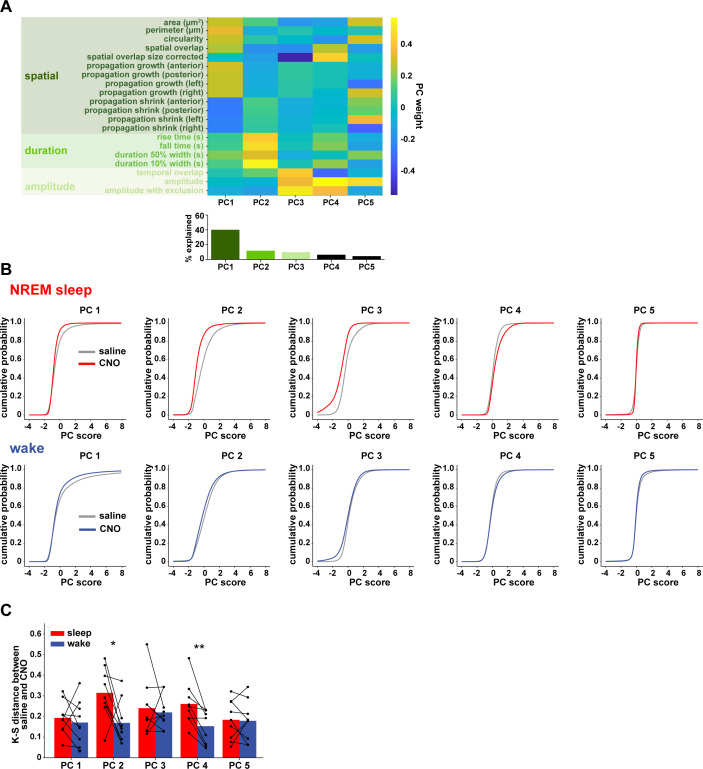
PCA shows that Gi-DREADD activation causes increased changes in Ca^2+^ event properties during sleep compared to wake. (**A**) Top: Weight matrix extracted from PCA, incorporating 20 Ca^2+^ event properties obtained with AQuA (n = 10 mice; 2 hr recordings; 204,172 events). PC 1 largely represents spatial features, PC 2 represents temporal features, and PC 3 represents features associated with amplitude. Bottom: Percent of variance explained by each PC. (**B**) Top: Cumulative distribution of the score for each PC for sleep events after saline or CNO administration, demonstrating clear differences in multiple PCs compared with wake (bottom). Bottom: Cumulative distribution of the score for each PC for wake events is similar between saline and CNO (n = 10 mice for saline, nine mice for CNO condition, 2 hr recordings; 33,882 sleep events; 153,646 wake events). (**C**) K-S distance between the saline and CNO distributions in panel B demonstrates significant differences between sleep and wake in the effect of CNO on PC 2, PC 4, and PC 5 (Wilcoxon signed rank test, n = 9 mice, median event number/mouse = 4139).

The same experimental setup as earlier (Figure 1A) was used, but mice were co-injected with *AAV-GFAP-GCaMP6f* and *AAV-GFAP-hM4D(Gi)-mCherry* to express both GCaMP6f and Gi-DREADD specifically in cortical astrocytes (Figure 3A–B, Figure 3—figure supplement 1). In these experiments, we monitored the effects of I.P. administration of the hM4Di agonist clozapine-N-oxide (CNO, 1 mg/kg) on Ca^2+^ dynamics, SWA, and sleep state. Because of the known sedative effects of CNO, we first verified that CNO itself (1 mg/kg, I.P) did not alter Ca^2+^ dynamics or sleep features in the absence of DREADD expression. We found no change in Ca^2+^ dynamics or sleep features between administration of 1 mg/kg CNO and the saline control (Figure 3—figure supplement 2). While Gi-DREADD has been used in astrocytes in vivo previously, its effects on astrocytic Ca^2+^ have not yet been established during natural wake and/or sleep. Here, we confirmed that Gi-DREADD activation indeed altered astrocyte Ca^2+^, causing an increase in event frequency across the entire 2 hr recording period after CNO administration (Figure 3C–D, Video 2). This finding is consistent with studies of astrocytic Ca^2+^ activity in ex vivo slices and in anesthetized mice (Durkee et al., 2019; Nagai et al., 2019; Chai et al., 2017). Next, we asked whether Gi-induced Ca^2+^ event increases were sufficient to alter SWA. We found that activation of Gi-DREADDs by CNO significantly increased SWA during sleep compared to a saline injection in the same animal (Figure 3E). In contrast, total time spent in sleep and wake was not affected by Gi-DREADD activation (Figure 3F,H). Thus, although the total duration of sleep did not change, the sleep was characterized by higher SWA, or greater sleep depth. Together, these data demonstrate that regulation of SWA and sleep duration can be separated, and that astrocyte Ca^2+^, through Gi-GPCR activation, is sufficient to increase SWA during sleep. We hypothesized that astrocytes were part of a homeostatic mechanism regulating SWA, where in response to decreases in SWA, astrocyte Ca^2+^ causes an increase in SWA. Here, we artificially increased Ca^2+^ beyond endogenous levels through Gi-GPCR signaling and found we could drive SWA increases above control levels, consistent with the hypothesis that astrocytes are part of a homeostatic mechanism that regulates SWA.

**Video 2.** Ca^2+^ dynamics following Gi-DREADD-activation. Example video of 20 min of elevated in vivo GCaMP activity in layer 2/3 V1 cortical astrocytes expressing Gi-DREADDs after 1 mg/kg CNO administration. Frame rate = 30 Hz. Scale bar = 50 μm.

Because we found similar relationships between endogenous Ca^2+^ dynamics and SWA in sleep and stationary wake (Figure 1H, Figure 2C–D), we next quantified the effect of Gi-GPCR activation on SWA during wake. In contrast to the change in SWA during sleep (Figure 3E), we found no change in SWA during the entire wake state (Figure 3G). Likewise, when calculating SWA only in the stationary wake state, we observed no significant difference in SWA (Figure 3—figure supplement 1f). This negative result suggests that a different mechanism underlies the astrocyte-SWA relationship in wake, and assigns the role of Gi-induced Ca^2+^ dynamics to regulating SWA specifically during sleep. To investigate this difference, we performed PCA on the Ca^2+^ data collected after saline or CNO administration. We found that CNO resulted in significantly larger differences in multiple PCs for sleep relative to wake (Figure 3—figure supplement 3). This selective change in Ca^2+^ event properties during sleep, but not wake, may explain the sleep-specific effects in SWA.

Because the astrocyte-SWA relationship is partly dependent on IP_3_R2s (Figures 1I and 2C–D), we tested whether the effect of Gi-GPCR activation on SWA was also dependent on IP_3_R2s by repeating these Gi-DREADD experiments in IP_3_R2 KO mice. Unlike control mice (Figure 3C–D), CNO administration did not significantly increase astrocyte Ca^2+^ in IP_3_R2 KO mice (Figure 3I), demonstrating that Gi-DREADD-induced Ca^2+^ events rely, at least in part, on IP_3_R2. In accordance with the lack of change in Ca^2+^ in the IP_3_R2 KO animals, we also observed no significant change in SWA with CNO administration (Figure 3J), indicating that the change in sleep depth we observe (Figure 3E) is dependent on IP_3_R2.

### Gi-DREADD astrocyte activation regulates delta waves more than slow oscillations

**Figure 4.**
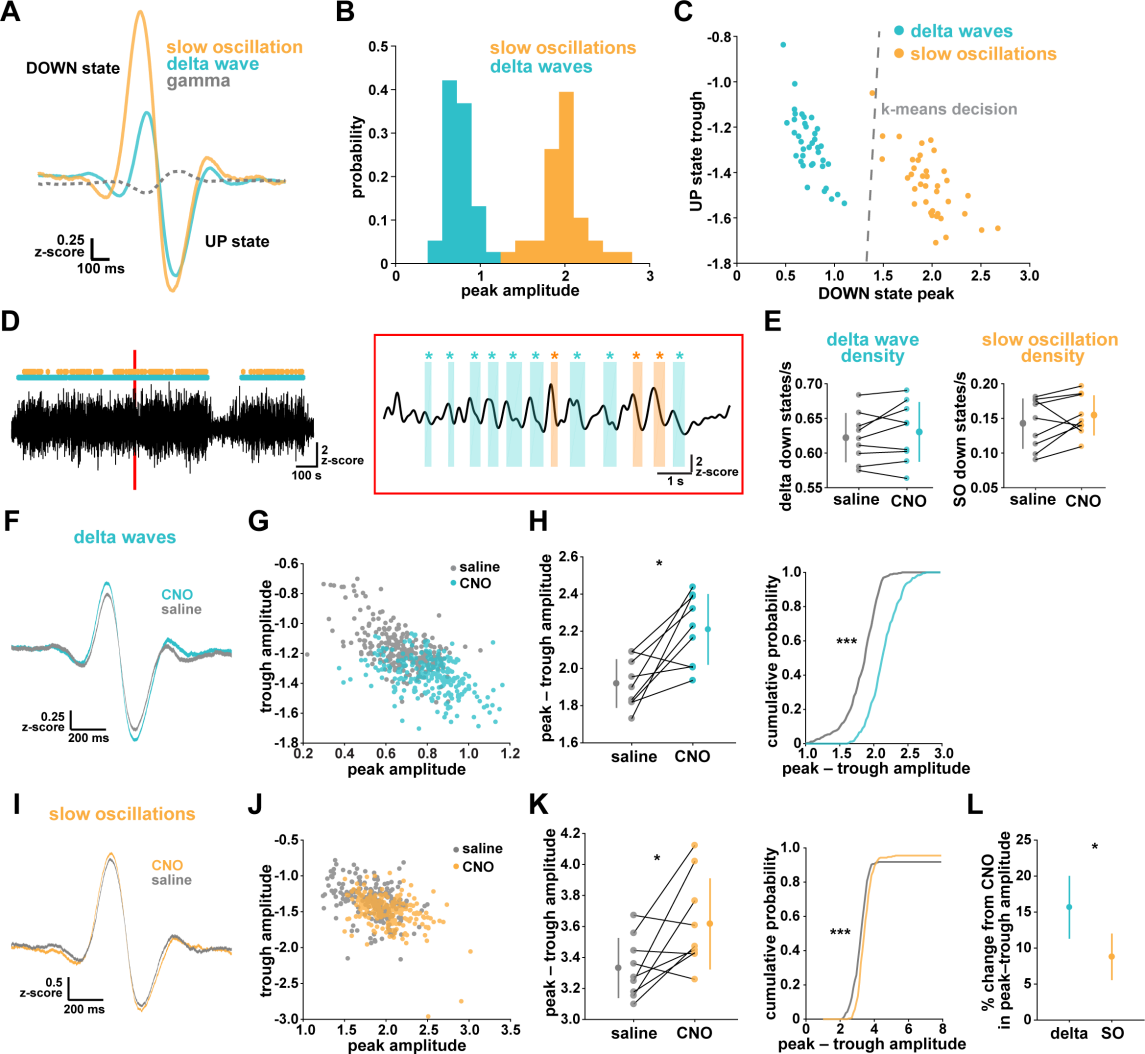
Astrocytic Gi-DREADD activation regulates local delta waves more than slow oscillations. (**A**) Mean of all identified slow oscillations (orange) and delta waves (cyan) (n = 31,966,966 delta waves, 7855 slow oscillations) in saline condition, including mean LFP amplitude filtered for high-gamma (gray, 80–100 Hz) demonstrating lower gamma during DOWN state and higher gamma during UP state (for panels **A–C**, n = 10 mice, 38 hr). (**B**) Peak amplitude separation between slow oscillations and delta waves. (**C**) Peak vs. trough amplitude for slow oscillations and delta waves is separable by K-means clustering (dashed line). (**D**) Left: Example of filtered LFP (0.1–4 Hz) for a 20-min recording. Slow oscillations (orange) and delta waves (cyan) indicated. Right: A 10 s window corresponding to the red box, with example waveforms of individual slow oscillations and delta waves. (**E**) Delta wave (left) and slow oscillation (right) rates do not change between conditions (for panels **E**; **H**, left; **K**, left; and **L**, data represented by mean for each animal, and the population as mean± SD, n = 9 mice 2 hr recordings, paired *t*-test). (**F**) Delta waves with CNO (cyan) show higher peak and trough amplitude compared with saline (gray) (saline: n = 10 mice, 16,467 waveforms; CNO: n = 9 mice, 15,499 waveforms). (**G**) Peak vs. trough amplitude for delta waves after CNO (cyan) is shifted compared with saline controls (gray) (for panels **G**; **H**, right; **J**; **K**, right, saline: n = 10 mice, 257 sleep periods, CNO: n = 9 mice, 246 sleep periods). (**H**) Left: Peak minus trough delta wave amplitude is higher with CNO. Right: Cumulative distribution reveals a leftward shift in the peak minus trough delta wave amplitude with CNO (two-sample Kolmogorov-Smirnov test). (**I**) Slow oscillations show minimal peak and trough amplitude change with CNO (orange) relative to saline (gray), compared with delta waves (**F**) (saline: n = 10 mice, 3995 waveforms, CNO: n = 9 mice, 3860 waveforms). (**J**) Peak versus trough amplitude for slow oscillations is similar between CNO (orange) and saline (gray) conditions, compared with delta waves (**G**). (**K**) Left: Peak minus trough slow oscillation amplitude shows a smaller, but significant, increase with CNO compared with delta waves (**H** left). Right: Cumulative distribution reveals a minimal shift in the peak minus trough slow oscillation amplitude after CNO administration, compared with delta waves (**H**, right) (two-sample Kolmogorov-Smirnov test). (**L**) Higher percent change for delta waves in peak minus trough amplitude with CNO, compared to slow oscillations.

While NREM sleep is broadly characterized by SWA, it has become increasingly clear that there are two main types of slow waves: delta waves and slow oscillations (Genzel et al., 2014; Kim et al., 2019; Steriade et al., 1993; Steriade and Timofeev, 2003; Siclari et al., 2014; Dang-Vu et al., 2008; Bernardi et al., 2018). These two types of slow waves are characterized by different regulatory mechanisms and are associated with distinct functions in NREM sleep. Delta waves are thought to promote the weakening of memories, while slow oscillations support memory consolidation (Genzel et al., 2014; Kim et al., 2019). In light of our finding that astrocytic Gi-GPCR-induced Ca^2+^ is sufficient to increase sleep SWA (Figure 3E), we explored whether this increase could be attributed to specific changes in delta waves or slow oscillations. A specific change could point to specific roles of astrocytic Gi-signaling in sleep. For this analysis, we implemented an established approach to distinguish delta waves and slow oscillations by their distinct waveforms (Kim et al., 2019). Slow oscillations had larger positive peaks and larger positive-to-negative deflections that occurred within 500 ms (Figure 4A–B). Across recordings, slow oscillations and delta waves were differentiated by their peak and trough amplitudes using k-means clustering (Figure 4C).

**Figure 4—figure supplement 1.**
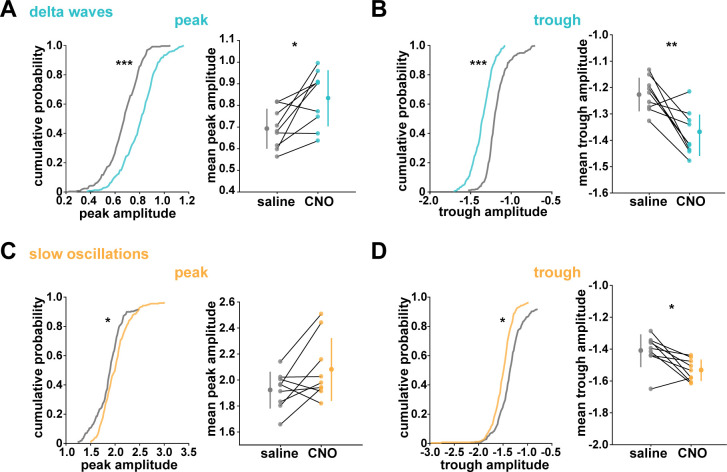
Gi-DREADD activation does not affect SWA in stationary wake, but changes peak and trough delta wave amplitude during sleep. (**A**) The cumulative distribution (left) and summary statistics across mice (right) show that delta wave peak amplitude is significantly higher with CNO compared to saline (for panels **A–D**, n = 10 mice, 38 total hrs for cumulative distribution plots, two-sample Kolmogorov-Smirnov test. n = 9 mice, 2 hr recordings/mouse, paired *t*-test for the remaining figures). (**B**) Similar to (**A**), cumulative distribution (left) and summary statistics across mice (right) reveal significantly lower trough amplitude after CNO administration relative to saline. (**C**) The cumulative distribution (left) shows a significant but smaller shift in slow oscillation peak amplitude with CNO compared to delta waves shown in (**A**). Summary statistics across mice show no significant change in slow oscillation peak amplitude between conditions. (**D**) Similar to (**C**), the cumulative distribution (left) and summary statistics across mice (right) demonstrate a significant, but smaller, change in slow oscillation trough amplitude compared with delta wave trough amplitude (**B**). For all panels with summary statistics, data represented by mean for each animal and population mean± SD.

We first looked at the effect of astrocytic Gi-DREADD activation on the number of identified delta waves and slow oscillations, and found no effect on the rate of delta waves or slow oscillations during sleep (Figure 4E). This negative result was expected by this analysis, because delta waves and slow oscillations were identified using amplitude percentile thresholds (see Materials and methods) that were set for each individual recording. However, when quantifying the amplitude of these waveforms, we noted increases in the mean amplitude, particularly for delta waves (Figure 4F,I). Indeed, by plotting peak vs. trough amplitude, we observed a clear change in delta waves after CNO, resulting in higher peak and lower trough amplitudes (Figure 4G). This change was smaller in the slow oscillation waveforms (Figure 4J). Similarly, we quantified the change in total peak – trough amplitude after CNO administration. While we saw a significant increase in size for delta waves (Figure 4H) and slow oscillations (Figure 4K) compared to saline controls in the same animal, the change in delta waves was significantly higher than that for slow oscillations (Figure 4L, Figure 4—figure supplement 1). Together, these data demonstrate that astrocyte Ca^2+^, through Gi-GPCR signaling, preferentially increases SWA by altering delta wave amplitude. Delta waves are more local than slow oscillations and are thought to be generated within the cortex (Genzel et al., 2014; Siclari and Tononi, 2017; Siclari et al., 2014; Bernardi et al., 2018; Spoormaker et al., 2010; Nir et al., 2011). Given that our Gi-astrocytic manipulation is restricted to a small portion of cortex (Figure 3—figure supplement 1), the result that astrocytic Gi-DREADD activation affects delta waves more than slow oscillations may indeed be expected.

### Cortical astrocyte Ca^2+^ dynamics exhibit bidirectional changes before sleep/wake transitions

**Figure 5.**
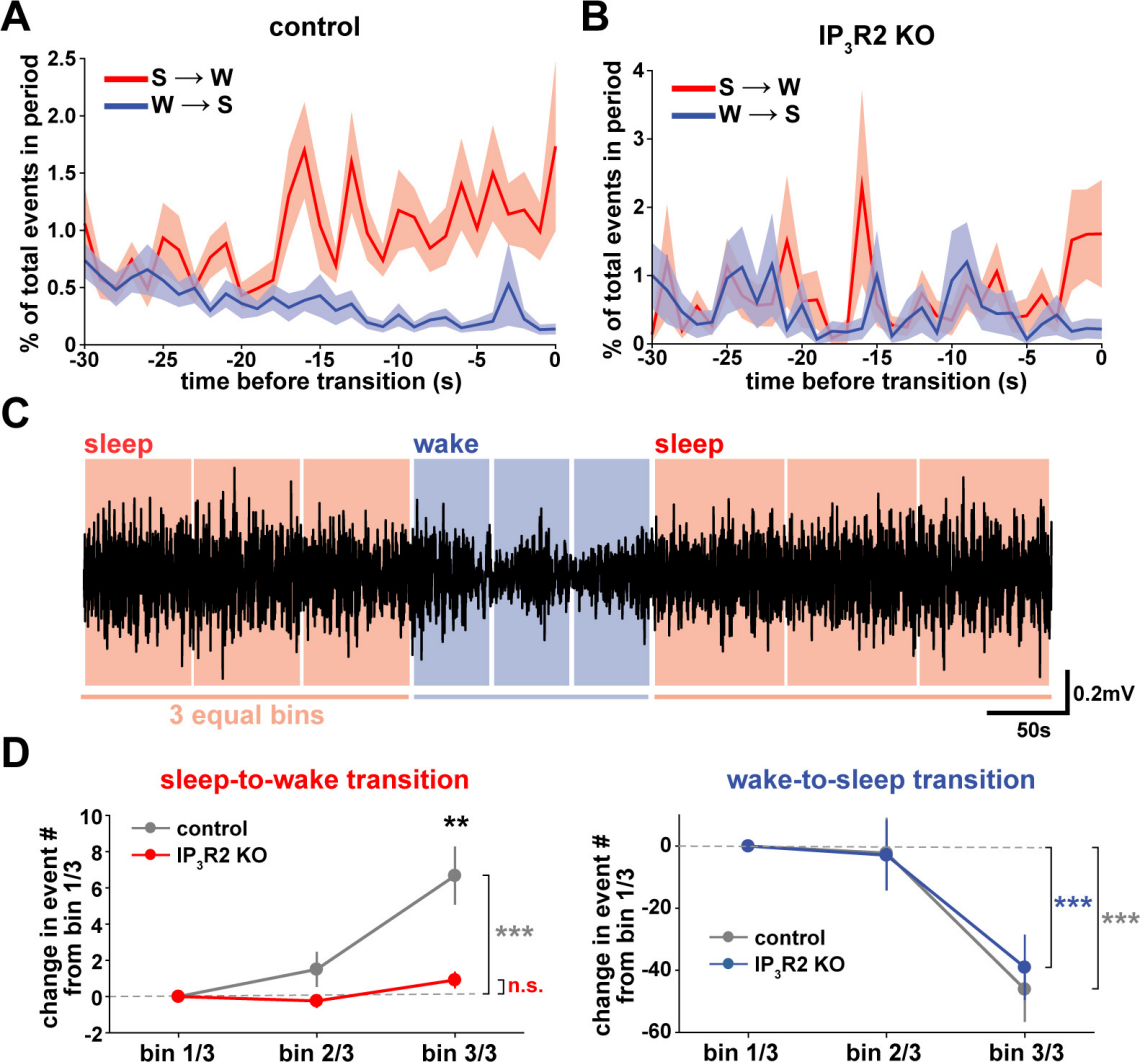
Cortical astrocyte Ca^2+^ dynamics exhibit bidirectional changes preceding sleep and wake transitions. (**A**) Within each individual sleep/wake period, the proportion of all astrocyte Ca^2+^ events increases before transitions to wake (red) and decreases before transitions to sleep (blue). Error bars=SEM. (**B**) In contrast, the proportion of all Ca^2+^ events in a sleep or wake period in IP_3_R2 KO mice does not consistently change preceding transitions. Error bars=SEM. (**C**) Example filtered LFP (0.5–4 Hz) trace across sleep (NREM) and wake transitions. All sleep (red) and wake (blue) periods were divided in three equal bins to examine Ca^2+^ dynamics across individual behavioral states. (**D**) Change in Ca^2+^ event number across sleep and wake periods show an increase in event number in the last third of sleep (left) and a decrease in the last third of wake (right) (paired *t*-test). Change in Ca^2+^ event number across sleep periods (left) increases in the last third for control, but not IP_3_R2 KO mice (unpaired *t*-test). Data are represented as mean± SEM (controls: n = 4 mice, 19 hr; IP_3_R2 KO = 5 mice, 22 hr).

We next wondered whether astrocytes might play a role beyond the regulation of sleep depth, to also influence sleep duration. Data here (Figure 2) suggest that a component of astrocyte signaling may be important for sleep/wake state transitions, which would directly affect sleep duration. To study these transitions, we first examined endogenous cortical astrocyte Ca^2+^ dynamics in the 30 s leading up to transitions between sleep or wake. We found a pattern in which Ca^2+^ events consistently increased before the sleep-to-wake transition and decreased before the wake-to-sleep transition (Figure 5A). This is supported by a recent study that demonstrated, using an alternative image analysis technique, that Ca^2+^ increases preceding sleep-to-wake transitions (Bojarskaite et al., 2020). We next divided all sleep and wake periods, regardless of length, into three equal bins (Figure 5C). This allowed us to study how astrocyte Ca^2+^ dynamics generally change throughout a sleep or wake period. In so doing, we found that Ca^2+^ event rate increased in the last third of sleep and decreased in the last third of wake (Figure 5D). Since Ca^2+^ event rate is higher during wake than sleep (Figure 1F) and Ca^2+^ events occur after dips in SWA (Figure 2C), the increase in event rate preceding the transition to wake could reflect a gradual shift in SWA to a wake state. In fact, various ascending brainstem neuromodulatory neurons associated with wakefulness have been shown to increase firing prior to the transition to wake and decrease firing prior to the transition to sleep (Takahashi et al., 2006; Eban-Rothschild et al., 2016; Aston-Jones and Bloom, 1981; Lee et al., 2005; Trulson and Jacobs, 1979). Astrocytes express receptors and exhibit increased Ca^2+^ dynamics in response to many of these neuromodulators (Ding et al., 2013; Khan et al., 2001; Takata et al., 2011; Shelton and McCarthy, 2000). Thus, this change in event rate prior to sleep/wake transitions may be due to neuromodulator-driven GPCR signaling in astrocytes.

We reasoned that if the Ca^2+^ dynamics observed around state transitions were due to astrocytic GPCR signaling, we would expect that these Ca^2+^ dynamics would be altered in IP_3_R2 KO mice. As predicted, we found that the changes in event rate preceding transitions were abolished in IP_3_R2 KO mice (Figure 5B). When quantifying the change in event rate in the last third of sleep and wake for IP_3_R2 KO mice, we found that IP_3_R2 KO mice did not exhibit the same increase in event rate in the last third of sleep (Figure 5D, left). However, the change in event rate observed during wake was unchanged in IP_3_R2 KO mice (Figure 5D, right), suggesting a specific role of IP_3_R2s in sleep. Since Gi-DREADD activation did not affect sleep duration (Figure 3F), we next tested the hypothesis that Gq-GPCR-mediated Ca^2+^ signaling in astrocytes regulates sleep/wake transitions.

### Gq-DREADD activation suppresses astrocyte Ca^2+^ dynamics in vivo

**Figure 6.**
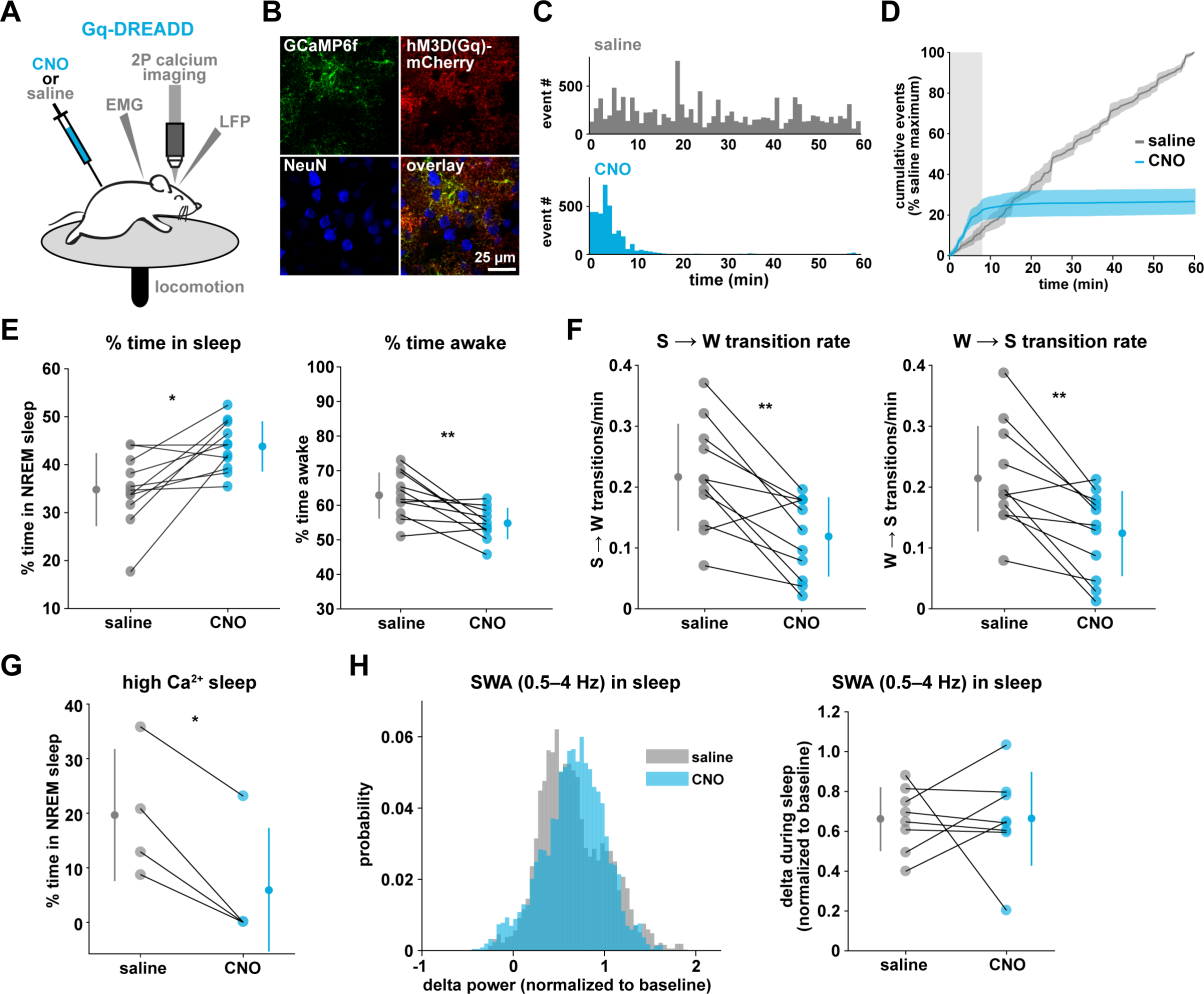
Gq-induced Ca^2+^ is necessary for sleep-wake transitions. (**A**) Experimental setup. Mice were co-injected with *GFAP-cyto-GCaMP6f* and *GFAP-hM3D(Gq)-mCherry* AAVs. After I.P. injection of either 1 mg/kg CNO or saline, 2P astrocyte Ca^2+^ dynamics, LFP, EMG, and locomotion were recorded. (**B**) Post-experiment immunohistochemistry demonstrates astrocyte-specific expression of the Gq-DREADD (red) and GCaMP6f (green). mCherry^+^ cells exhibit typical astrocyte morphology and do not co-localize with neurons (blue, NeuN). (**C**) Representative data from a single Gq-DREADD-expressing animal. Administration of CNO (blue) causes a short initial period of elevated Ca^2+^ relative to saline administration (gray), followed by a complete suppression of all Ca^2+^ activity. (**D**) Cumulative Ca^2+^ event count after saline (gray) or CNO (blue) injection over 60 min. The initial high Ca^2+^ period (8 min, light gray box) is followed by suppression of astrocyte Ca^2+^. (Error bars=SEM, n = 3 mice, 1 hr recordings) (**E**) Left: The proportion of time mice spend sleeping after CNO administration (during Ca^2+^ suppression period) is increased relative to saline controls, and time in wake is decreased (right), suggesting Ca^2+^ suppression is sufficient to increase sleep (for E–F, and H, paired *t*-test, n = 11 mice; analyses are performed in the 1 hr, 52-min period of Ca^2+^ suppression). (**F**) Sleep-to-wake transitions (left) and wake-to-sleep transitions (right) are decreased with CNO relative to saline. (**G**) During the high Ca^2+^ period after CNO administration, mice spend less time sleeping compared to saline-injected controls (paired *t*-test), suggesting the Gq-DREADD-driven Ca^2+^ increase is sufficient to suppress sleep (n = 4 mice, for E–H, data are represented as mean for each animal and population mean± SD). (**H**) Distribution of SWA (left) and summary statistics (right) show that despite Ca^2+^ changes, SWA during sleep is unaffected by Gq-DREADD activation. (n = 8 mice, paired *t*-test).

To drive the astrocytic Gq-GPCR pathway and test for a role of astrocyte Ca^2+^ in mediating sleep/wake transitions, we selectively expressed the human M3 muscarinic receptor DREADD (hM3Dq) in astrocytes. We were also motivated by the knowledge that neuromodulatory signals play an important role in mediating sleep and wake transitions (Holst and Landolt, 2018; Saper and Fuller, 2017; Lee and Dan, 2012; Scammell et al., 2017), and many of these endogenous signals can act at Gq-GPCRs in astrocytes (Zhang et al., 2014; Chai et al., 2017). We used a similar approach as above (Figure 3A), but here selectively expressed GCaMP6f and the Gq-DREADD in astrocytes (Figure 6A–B, Figure 6—figure supplement 1). As above, we imaged astrocyte Ca^2+^ after I.P. CNO administration to confirm the effect of Gq-DREADD activation on Ca^2+^ activity in vivo. Although astrocytic Gq-DREADD activation in vivo has been performed previously (Durkee et al., 2019; MacDonald et al., 2020; Bonder and McCarthy, 2014; Adamsky et al., 2018), validation of Gq-DREADD-mediated astrocytic Ca^2+^ increases has only been performed under anesthesia or ex vivo, in part because several in vivo astrocyte DREADD experiments have been carried out in brain regions that are less accessible than cortex (MacDonald et al., 2020; Adamsky et al., 2018). Thus, the effect of Gq-DREADD activation on Ca^2+^ in awake mice has not been previously reported. Canonically, Gq-GPCR signaling results in an increase in Ca^2+^ activity via IP_3_-dependent release of intracellular Ca^2+^ (Petravicz et al., 2008). However, we were surprised to find that Ca^2+^ dynamics only increased in the first 5–10 min after I.P. injection of CNO (150.9% ± 135.9, Video 3). After this initial period of increased Ca^2+^ events, Ca^2+^ dynamics were almost completely abolished (−97.3% ± 0.79%, Figure 6C–D, Figure 6—figure supplement 1H, Video 3). This ‘silent’ state of Ca^2+^ dynamics lasted for the rest of the entire recording (2–3 hr).

**Figure 6—figure supplement 1.**
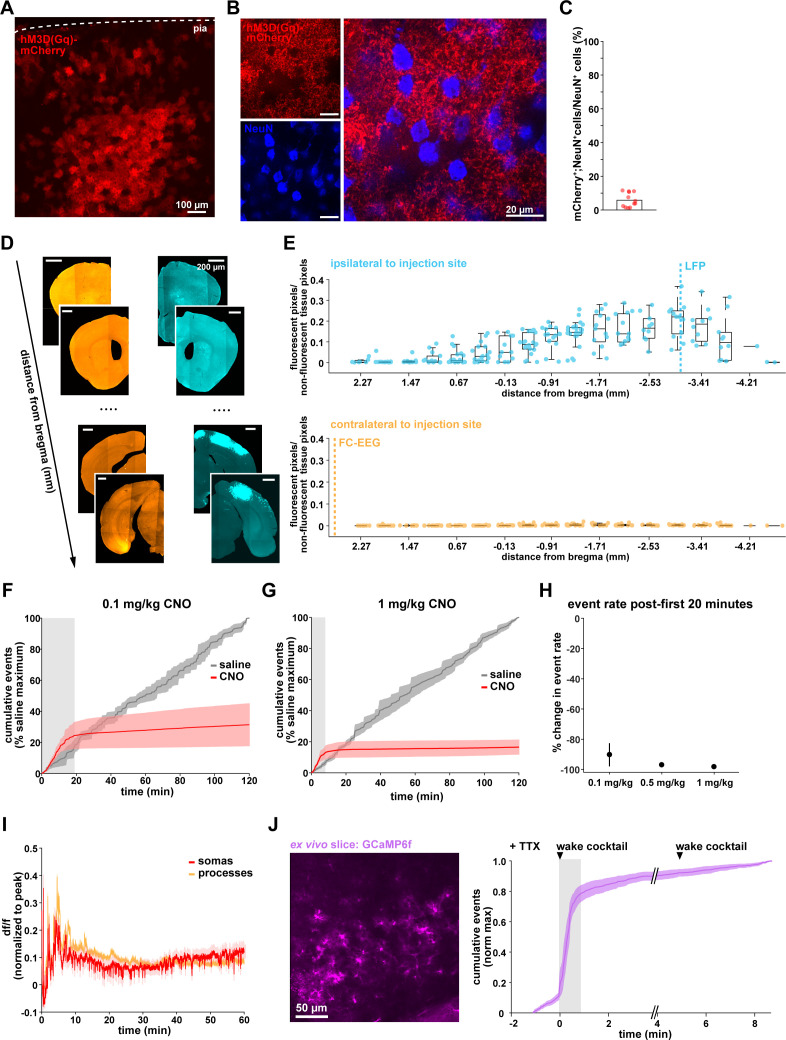
Astrocyte-specific expression of Gq-DREADDs across ipsilateral and contralateral cortex. (**A**) Representative post-experiment immunohistochemistry demonstrating astrocyte-specific expression of Gq-DREADDs across cortical layers. (**B**) Representative 63x confocal images of mCherry and NeuN staining to quantify non-specific DREADD expression. (**C**) Percentage of NeuN^+^ identified cells that were also mCherry^+^. (**D**) Schematic illustrating analysis of cortex-wide Gq-DREADD expression spread in hemispheres both ipsilateral and contralateral to viral injection site. (**E**) Expression spread in brain slices sampled from rostral to caudal for the ipsilateral (top, cyan) and contralateral hemisphere (bottom, orange) shows that expression is highest in ipsilateral rostral cortex, near the LFP recording site (dashed line, cyan). No expression is detected at the FC-EEG recording site (dashed line, yellow). Viral expression spread for each slice was calculated by normalizing the number of fluorescent pixels to the size of the brain tissue. Data represented with boxplots marking the medians, 25th and 75th percentiles. (**F**) Cumulative Ca^2+^ event count for mice after administration of 0.1 mg/kg CNO (red) and saline (gray) showing an initial period of elevated Ca^2+^ (gray box), followed by a dramatic suppression of Ca^2+^ events similar to 1 mg/kg CNO (Error bars=SEM, n = 3 mice, 1 hr recordings). (**G**) Cumulative Ca^2+^ event count for mice after 1 mg/kg CNO (red) and saline (gray), as shown in Figure 5D (Error bars=SEM, n = 3 mice, 1 hr recordings). (**H**) Total suppression of astrocyte Ca^2+^ event rate is similar for all three tested doses of CNO (mean± SEM, 0.1 mg/kg: n = 3 mice, 2 hr recordings; 0.5 mg/kg: n = 1 mouse, 2 hr recordings; 1 mg/kg: n = 5 mice, 2 hr recordings). (**I**) Mean GCaMP6f fluorescence in somata and processes after administration of 1 mg/kg CNO, with both remaining elevated throughout 1 hr recording (Error bars=SEM, n = 3 mice, 1 hr recordings). (**J**) Left: Representative 2P image of V1 cortical astrocytes in an acute, ex vivo slice. Right: Cumulative events across two consecutive 5 min videos demonstrating a large response to the first application of a ‘wake’ cocktail (black triangles), but not the second (error bars=SEM, n = 2 mice, eight slices).

**Figure 6—figure supplement 2.**
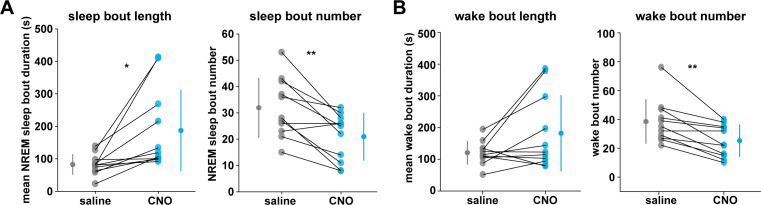
Gq-DREADD activation suppresses sleep-wake transitions by increasing bout length and decreasing bout number. (**A**) Left: Mean sleep bout length after CNO administration is increased compared to saline controls, suggesting Ca^2+^ dynamics are necessary for sleep-to-wake transitions. Right: Mean sleep bout number observed after CNO administration is decreased compared to saline controls. For all panels, n = 11 mice. Analyses are performed during the 1 hr, 52 min period when Ca^2+^ is suppressed, all paired *t*-tests. (**B**) Left: Mean wake bout length after CNO administration is increased relative to saline, suggesting Ca^2+^ dynamics are necessary for wake-to-sleep transitions, similar to sleep-to-wake transitions in (**A**). Right: Mean number of wake bouts after CNO, and during Ca^2+^ suppression, is decreased relative to saline.

**Video 3.** Ca^2+^ dynamics following Gq-DREADD-activation. Example video of 20 min of in vivo GCaMP activity in layer 2/3 V1 cortical astrocytes expressing Gq-DREADDs. Video begins immediately after 1 mg/kg CNO administration, demonstrating the initial period of elevated Ca^2+^, followed by almost complete Ca^2+^ suppression. Frame rate = 30 Hz. Scale bar = 50 μm.

To test whether this unexpected result was due to CNO concentration (1 mg/kg), we administered lower doses of CNO. While the initial period of increased Ca^2+^ dynamics was slightly longer (15–20 min) following administration of a ten-fold lower dose of CNO (0.1 mg/kg), this very low dose still resulted in a strong reduction in Ca^2+^ events for long time periods (Figure 6—figure supplement 1F–h). The observed inhibition of astrocyte Ca^2+^ could be due to depletion of intracellular Ca^2+^ stores and/or interference with store-operated Ca^2+^ channels (Sakuragi et al., 2017). To compare the inhibition of Ca^2+^ events with changes in fluorescence, we used an ROI-based approach to analyze fluorescence in somas and processes after CNO administration. We found that fluorescence in both somas and processes remained elevated above baseline after 1 mg/kg CNO (Figure 6—figure supplement 1I), which suggests that Ca^2+^ levels may be clamped at saturating levels. Together, these results indicate that (1) we cannot assume that Gq-DREADD activation simply increases astrocytic Ca^2+^ in vivo, and (2) when feasible, astrocytic experiments using chemogenetics in vivo should be validated individually, particularly for those involving circuit function and animal behavior.

To test whether the astrocytic Ca^2+^ silencing we observed following Gq-DREADD activation could be reproduced with endogenous GPCR signaling, we measured Ca^2+^ activity in ex vivo cortical slices in response to a cocktail of neuromodulators associated with wakefulness, including norepinephrine, acetylcholine, dopamine, and histamine. We adapted methodology (Ding et al., 2016), using half the concentration of each neuromodulator as previously, since each experiment involved two total applications of this ‘wake cocktail’ (20 μM norepinephrine, 5 μM acetylcholine, 5 μM dopamine, 2.5 μM histamine). We also included TTX in the circulating bath to block neuronal firing. As predicted from previous studies reporting astrocytic Ca^2+^ increases to various neuromodulators (Ding et al., 2013; Khan et al., 2001; Takata et al., 2011; Shelton and McCarthy, 2000; Pankratov and Lalo, 2015), we observed a dramatic increase in Ca^2+^ activity in response to the cocktail (Figure 6—figure supplement 1J). However, after this initial increase in Ca^2+^, GCaMP fluorescence did not return to baseline levels, but remained high and further Ca^2+^ events were almost completely absent (Figure 6—figure supplement 1J), similar to in vivo dynamics observed 5–10 min after CNO administration. To test whether this ‘silent’ state altered the astrocytic response to further neuromodulatory input, we bath-applied a second round of the wake cocktail. In contrast to the initial Ca^2+^ increase, we observed no further increase in astrocyte Ca^2+^ (Figure 6—figure supplement 1J). We speculate that the mechanism underlying the inability of astrocytes to respond to a second dose of wake cocktail may be similar to that underlying the inhibition of Ca^2+^ dynamics in vivo in response to circulating CNO.

### Gq-induced Ca^2+^ dynamics regulate sleep-wake transitions

The finding that Gq-DREADD chemogenetics can inhibit an intracellular GPCR signaling pathway in astrocytes makes this is a particularly useful tool for understanding astrocytes' roles in cortical state regulation. To investigate whether astrocytes regulate sleep duration, we focused on the long period of Ca^2+^ suppression in these experiments. We found that mice spent significantly more time in sleep after CNO administration (Figure 6E, left). Further, in the absence of Gq-GPCR-mediated Ca^2+^ events, mice made fewer sleep-to-wake transitions (Figure 6F, left) and accordingly, we observed fewer sleep bouts of longer duration (Figure 6—figure supplement 2A). This suggests that the IP_3_R2-dependent increase in event rate prior to sleep-to-wake transitions (Figure 5) is important to transition the cortex to the wake state. The transition data (Figure 5) also showed that endogenous Ca^2+^ decreases toward the end of wake periods, just prior to wake-to-sleep transitions (Figure 5). Thus, we wondered whether Ca^2+^ suppression via Gq-DREADDs would affect wake as well. We observed a decrease in the percent time awake (Figure 6E, right), as predicted by the increase in sleep observed (Figure 6E, left). However, we also observed less frequent transitions out of wake, demonstrating that astrocyte Ca^2+^ is important for both wake-to-sleep transitions (Figure 6F, right), and sleep-to-wake transitions (Figure 6F, left). As predicted from the decrease in transitions, we also observed fewer wake bouts and wake bouts of longer duration (Figure 6—figure supplement 2B). We hypothesize that decreased astrocytic Ca^2+^ prior to wake-to-sleep transitions (Figure 5) is important for the transition to sleep, but astrocytes were unable to make this significant decrease due to clamped Ca^2+^ in these experiments.

If Gq-GPCR signaling is an important bidirectional regulator of sleep/wake transitions, we would expect that increases in Gq-GPCR signaling to have the opposite effect from decreased Gq-GPCR Ca^2+^ signaling. We thus used data from the short, initial period with elevated Ca^2+^ activity to ask whether this is the case. Because this period is so short (5–10 min), we were somewhat limited in our analysis. However, of the animals that exhibited some sleep in either the CNO or saline condition (n = 4), we observed a significant decrease in the percent time sleeping (Figure 6G). This bidirectional change in sleep time strongly supports the hypothesis that Gq-GPCR-mediated Ca^2+^ plays a critical role in regulating sleep duration. Interestingly, we did not observe a change in the amount of sleep with Gi-GPCR activation (Figure 3F), which similarly increased Ca^2+^ dynamics. This difference between Gq- and Gi-mediated Ca^2+^ increases indicates an important functional dissociation between Gq- and Gi-GPCR-mediated Ca^2+^ activity in astrocytes and highlights the likelihood that other signaling molecules involved in GPCR signaling cascades play roles in regulating sleep-wake transitions. Because we observed a significant increase in sleep depth in response to the Ca^2+^ increase with Gi-DREADDs (Figure 3E), we also wondered whether Ca^2+^ suppression via Gq-DREADDs would have an opposing effect. In contrast to manipulation of the Gi-GPCRs, we found that Ca^2+^ suppression via Gq-GPCR manipulation had no significant effect on SWA during sleep (Figure 6H). This suggests that astrocytic regulation of SWA is specifically dependent on the Gi-GPCR pathway and provides further evidence that astrocytic Gi- and Gq-GPCR signaling regulate separable sleep/wake features.

### Local Gi-DREADD activation of cortical astrocytes can drive changes in remote cortical sleep features

**Figure 7.**
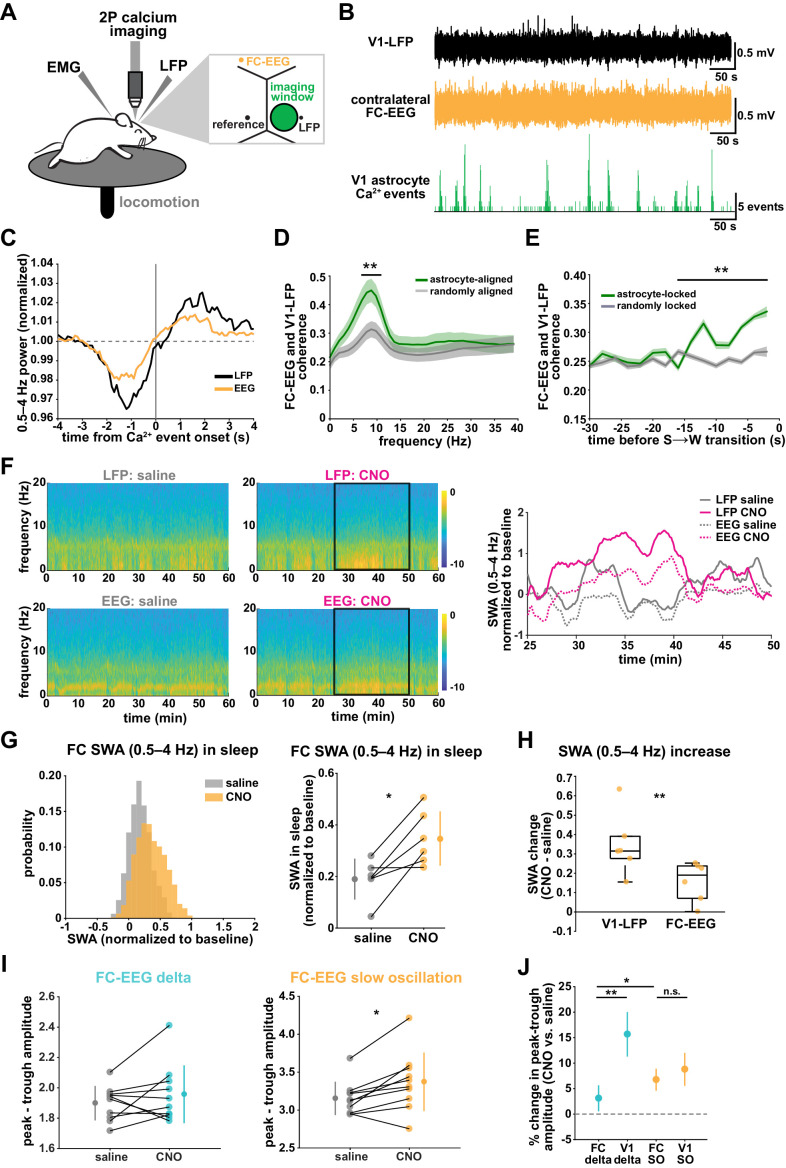
V1 manipulation of astrocyte Ca^2+^ alters SWA in contralateral frontal cortex via changes in slow oscillations. (**A**) Experimental setup. Mice were co-injected with *GFAP-cyto-GCaMP6f* and *GFAP-hM4D(Gi)-mCherry* AAVs. 2P astrocyte Ca^2+^ dynamics in right V1, LFP local to the imaging field, EEG in the contralateral frontal cortex (orange), EMG, and locomotion were recorded. (**B**) Representative data from simultaneous recordings from V1-LFP (black), contralateral frontal cortex EEG (orange), and V1 astrocyte Ca^2+^ imaging (green). (**C**) Average V1-Ca^2+^ event-triggered traces of FC-EEG SWA (orange) reveal a similar, but smaller, fluctuation of SWA around Ca^2+^ event onsets compared with V1-LFP (black). (**D**) Coherence between V1-LFP and FC-EEG is higher after astrocyte event onset (green), compared to randomly sampled time points (gray) (paired *t*-test, n = 13 mice, median event number/mouse = 7512,512 events). Error bars=SEM. (**E**) Astrocyte-locked coherence (0–15 Hz) increases before sleep-to-wake transitions (unpaired *t*-test, n = 13 mice, median event number/mouse = 417 events). (**F**) Left: Example spectrograms from simultaneously recorded population-level electrophysiology: V1-LFP (top row) and FC-EEG (bottom row) following saline (left column) or CNO (right column) injection. Right: SWA corresponding to the black rectangle marked in the spectrograms demonstrates that CNO (pink) increased SWA in V1-LFP (solid lines) and FC-EEG (dashed lines) compared to saline (gray). (**G**) Distribution of SWA (left) and summary statistics across mice (right, paired *t*-test) demonstrate that SWA in FC-EEG is increased after activation of V1 Gi-DREADD-expressing astrocytes by CNO (data represented as mean for each animal and population mean± SD, n = 7 mice, 2 hr recordings). (**H**) Change in SWA measured by V1-LFP or FC-EEG demonstrate that CNO increases SWA in both measurements, but more in V1-LFP recordings (paired *t*-test). Data represented using box plots with median, 25th and 75th percentile. (**I**) Peak-to-trough amplitude for delta waves (cyan, left) and slow oscillations (yellow, right) in saline (gray) and CNO conditions in FC (paired *t*-test). (**J**) Percent change in peak-trough amplitude for delta waves (cyan) and slow oscillations (yellow) in CNO vs. saline (paired *t*-test, data represented as mean± SEM). There is a greater change in amplitude for slow oscillations than delta waves in FC after Gi-DREADD activation in V1.

SWA during NREM sleep is considered a widespread phenomenon, involving the synchronization of neurons across the entire cortex. While widespread oscillatory activity has been observed in several animal models (Lemieux et al., 2015; Amzica and Steriade, 1995), recent work has also emphasized the existence of more local and asynchronous sleep (Genzel et al., 2014; Huber et al., 2004; Funk et al., 2016; Siclari and Tononi, 2017; Bernardi et al., 2018; Nir et al., 2011). The morphology and interconnectedness of cortical astrocytes and astrocytic networks make them well positioned to mediate neural activity across broad swaths of cortex. Cortical astrocytes are non-overlapping, in all cortical layers, gap junctionally coupled, and contain highly ramified processes that can contact tens of thousands of synapses (Halassa et al., 2007). We therefore wondered how they may be involved in both local and remote changes in cortical synchronization in sleep. To address this question, we implanted a second electrode to record EEG in the contralateral frontal cortex (FC-EEG, Figure 7A). This second electrode was far (both rostral-caudally and medial-laterally) from the imaging window/LFP electrode in V1, but still over cortex (Figure 7A, Figure 3—figure supplement 1, Figure 6—figure supplement 1).

### Astrocyte Ca^2+^ dynamics are associated with changes in coherence between V1 and contralateral FC

With two recording sites, we first explored endogenous relationships between astrocytic Ca^2+^ events in V1 and cortical state in contralateral FC in sleep. Using Ca^2+^ event-triggered averages, we found a similar relationship with V1 Ca^2+^ events and FC as previously described (Figure 2C): SWA in FC decreased before and increased after V1 Ca^2+^ event onsets, although the magnitude of this modulation was smaller than that observed locally (Figure 7C). To look at the synchronization between these cortical areas, we examined the coherence between local V1 and remote FC oscillations. We found that the V1-FC coherence (between 5 and 10 Hz) was higher immediately following astrocyte Ca^2+^ events when compared to coherence measured from randomly chosen epochs (Figure 7D). We also found an increase in astrocyte event-locked coherence (0–15 Hz) at the end of sleep periods, in the 15 s prior to sleep-to-wake transitions (Figure 7E). These data provide evidence that astrocytes may be involved in mediating endogenous cortex-wide physiological activity.

### Local cortical astrocyte activation increases SWA and slow oscillations in remote cortex

We next tested whether astrocytes play a causal role in brain-wide SWA during sleep using Gi-DREADD activation in V1 and the FC-EEG. To assess how the spread of astrocytic DREADD expression compared with the location of the two recording electrodes, we performed immunohistochemistry on brain slices across the rostral-caudal axis (Figure 3—figure supplement 1D). As expected, the majority of expression was centered around the V1-LFP electrode where viruses had been injected, while no expression was observed in FC at the site of the EEG electrode (Figure 3—figure supplement 1E). To assess a causal role for astrocyte Ca^2+^ in brain-wide oscillatory activity, we compared the effect of Gi-DREADD activation of V1 astrocytes at both V1-LFP and FC-EEG electrodes. Here, we found that increasing Ca^2+^ via Gi-DREADDs in V1 was sufficient to increase SWA in the contralateral frontal cortex, although this increase was smaller than that observed in V1 (Figure 7F–H). We also found that the SWA change was accounted for by a significant increase in slow oscillation amplitude, but not delta wave (Figure 7I; Figure 7J). This is in contrast with the greater delta wave change observed locally (Figure 4I). Moreover, the change in slow oscillation amplitude in FC (Figure 7J, 6.8 ± 2.2%) was similar to the change of the slow oscillation amplitude in V1 (Figure 4L, 8.8 ± 3.2%), suggesting that slow oscillations generated in V1 travelled to FC. These results indicate that astrocytes can influence cortex-wide dynamics on a large scale via specific changes to the slow oscillation component of SWA.

## Discussion

Using simultaneous 2P imaging of astrocyte Ca^2+^ and electrophysiology across wake and sleep, we have demonstrated that cortical astrocytes regulate distinct features of sleep via differential GPCR signaling. We showed IP_3_R2-dependent Ca^2+^ activity is inversely correlated with SWA and changes bidirectionally prior to sleep-wake transitions. With chemogenetics, we demonstrated that astrocyte Ca^2+^ is sufficient to increase SWA during sleep through specific enhancement of delta waves. This is specific to Gi-, not Gq-, GPCR-induced Ca^2+^. In contrast, Gq-, but not Gi-, GPCR Ca^2+^ signaling is important for mediating sleep/wake transitions. Lastly, we demonstrated that astrocyte Ca^2+^ has far-reaching, cortex-wide effects on SWA and slow oscillations.

### Sleep depth and sleep length are separable

We found that astrocytic Gi-DREADD activation increases SWA, but not sleep duration, while Gq-DREADD activation altered sleep duration but not SWA. This suggests a separation in the mechanisms underlying sleep depth, measured by SWA, and sleep duration. Previous work has also shown separation in sleep depth and duration by demonstrating that following sleep deprivation, recovery sleep has higher sleep depth but the duration is not significantly changed (Dijk and Beersma, 1989; Patrick and Gilbert, 1896). On the other hand, Ca^2+^ has been suggested to mechanistically link the regulation of sleep duration and depth (Ode et al., 2017; Tatsuki et al., 2016). Interestingly, our data supports both these ideas. We found that endogenous astrocyte Ca^2+^ is modulated in relation to both sleep duration (Figure 5) and sleep depth (Figure 1). Nevertheless, we were able to affect one without the other by selectively manipulating different GPCR pathways, suggesting these mechanisms are also separable.

The two-process model of sleep regulation has attributed the regulation of sleep duration to the interaction of sleep pressure, measured by SWA, and circadian rhythm (Borbély et al., 2016; Daan et al., 1984; BorbelyBorbély, 1982). Our findings that Gi-DREADD activation increased SWA without affecting sleep duration (Figure 3E,F) suggests that SWA does not directly influence sleep duration. However, we cannot discount the two-process model from this data alone, since we did not investigate the effect of circadian rhythm, nor did we directly study sleep homeostasis; our recordings were performed at the same time of day and were limited to 2–3 hr. In fact, astrocyte Ca^2+^ changes with circadian rhythm in the suprachiasmatic nucleus (Brancaccio et al., 2017) and increases with sleep need after sleep deprivation (Ingiosi et al., 2020). Longer recordings and sleep deprivation interventions to examine astrocytic integration of circadian signals and sleep pressure will be informative. Additionally, our methodology led us to focus on the role of astrocyte GPCR signaling in NREM sleep regulation, although previous work has demonstrated astrocyte Ca^2+^ changes with REM sleep (Bojarskaite et al., 2020; Ingiosi et al., 2020; Foley et al., 2017). The further study of astrocytic regulation of REM sleep may reveal interesting differences between regulation of behavioral sleep and cortical state, which is similar between REM sleep and wake. Indeed, our data suggests that Gi-GPCR signaling would be much attenuated during REM sleep, which is characterized by a lack of SWA.

Since astrocytes differentially control SWA and sleep/wake transitions, we hypothesize that Gi- and Gq- GPCR activation in astrocytes—while both drive Ca^2+^ changes—lead to different downstream effects which may elucidate new mechanisms of sleep regulation. In fact, many astrocytic functions associated with sleep may be important, such as extracellular glutamate regulation (Poskanzer and Yuste, 2016), extracellular ion dynamics (Ding et al., 2016), and adenosine release (Halassa et al., 2009). While we don’t yet know what downstream astrocytic effects underlie the sleep changes observed here, we have established that the functional astrocytic output is a not a simple consequence of changed Ca^2+^ levels in the cell, but rather of signaling downstream of either Gi- or Gq-GPCRs. Many new optical sensors, such as those for glutamate (Marvin et al., 2013), ATP (Lobas et al., 2019; Kitajima et al., 2020), and adenosine (Wu, 2020), in combination with astrocyte-specific manipulations, may be useful to link specific GPCR-driven Ca^2+^ dynamics with relevant astrocyte outputs.

### Functional dissociation between Gi- and Gq-GPCR Ca^2+^ signaling in astrocytes

The activation of both astrocytic Gi- and Gq-GPCRs increases intracellular Ca^2+^ (Durkee et al., 2019; Mariotti et al., 2016; Nagai et al., 2019), and yet we observed a functional dissociation between manipulation of the Gi- and Gq-GPCR pathways. These data underscore the complexity of Ca^2+^ signals in astrocytes and demonstrate that caution is necessary when attributing astrocytic Ca^2+^ increases to one specific downstream function. Many different roles have been attributed to astrocytes, suggesting astrocytes have the capacity to perform several functions in parallel. Our findings suggest that, through GPCR signaling, astrocytes interpret Ca^2+^ dynamics within the cell differently, resulting in different functional outputs. This may be a consequence of the many other signaling molecules downstream of Gi- and Gq-GPCRs, including phospholipase C or protein kinase A. Tools such as AQuA (Wang et al., 2019) that allow the accurate capture of complex astrocyte Ca^2+^ signaling are a first step in elucidating how Ca^2+^ dynamics map to the myriad of functions associated with astrocytes. The next step we took was to extract meaning from these signals by analyzing the multi-dimensional nature of astrocyte Ca^2+^ signals. We used PCA to reduce the 20 different properties describing each Ca^2+^ event and revealed differences that were not observed with individual comparisons of event rate, duration, size, or amplitude (Figure 1—figure supplement 1, Figure 3—figure supplement 3). This both illustrates the complexity of astrocyte Ca^2+^ signaling and emphasizes the importance of implementing more robust analysis tools.

Understanding how Gi- and Gq-GPCR activation gives rise to different effects on sleep will require further examination of these signaling pathways in astrocytes. First, we will need to identify the specific endogenous ligands during sleep that alter SWA and sleep/wake transitions. Candidates for sleep/wake transitions include neuromodulators, since many Gq-GPCRs for neuromodulators are expressed by astrocytes. In contrast, regulation of SWA has been attributed to both GABA (Lemieux et al., 2015; Sheroziya and Timofeev, 2014; Zucca et al., 2017) and glutamate (Sanchez-Vives and McCormick, 2000; Poskanzer and Yuste, 2011). GABA and glutamate are attractive candidate endogenous ligands because astrocytic GABAergic (via GABA_B_) and glutamatergic (via mGluR3 in adults) signaling are both mediated via Gi-GPCRs. Acute, astrocyte-specific knock-out of these receptors will provide important insight into the relevant receptors. Importantly, astrocyte-specific knock-outs will also reveal whether Gi-GPCR signaling is necessary to regulate SWA. While we demonstrated that astrocytic Gi-GPCR signaling is sufficient to alter SWA, other signaling cascades and cell types may also play important roles in SWA regulation. Second, further studies will be required to understand how intracellular signaling cascades for Gi- and Gq-GPCRs differ in astrocytes. Using PCA, we found differences in the effect of Gi-DREADD activation on Ca^2+^ in sleep versus wake, suggesting the action of the Gi-DREADD may be interacting with endogenous signaling that differs across behavioral states. Both Gi- and Gq-GPCRs increase Ca^2+^ via IP_3_R2 (Durkee et al., 2019), and we similarly noted a partial dependence on IP_3_R2 in both Gi- and Gq-GPCR-mediated sleep effects (Figures 3 and 5). This only partial dependence on IP_3_R2s in this data could be due to compensation for global IP_3_R2 absence, but it could also indicate that perhaps other signaling molecules unique to Gq- and Gi-signaling are critical for the sleep features described here. In fact, 1.4% of all astrocyte transcripts are regulated by sleep/wake state (Bellesi et al., 2015) and multiple biochemical assays have already identified various important molecules in sleep/wake regulation (Suzuki et al., 2013; Funato et al., 2016; Mikhail et al., 2017). Similar molecular studies specifically focused on astrocytic GPCR signaling will be critical to further understand the regulation of sleep duration and depth.

### Chemogenetic activation of astrocytes

While astrocytes have previously been implicated in sleep physiology (Bellesi et al., 2015; Ding et al., 2016; Halassa et al., 2009; Papouin et al., 2017; Petit and Magistretti, 2016; Bellesi et al., 2018; DiNuzzo and Nedergaard, 2017; Bojarskaite et al., 2020; Ingiosi et al., 2020; Foley et al., 2017; Ulv Larsen et al., 2020; Frank, 2013; Clasadonte et al., 2017), we present the first example of an acute in vivo astrocytic manipulation that changes natural sleep. Acute manipulation via chemogenetics was advantageous because DREADD activation mimics endogenous signaling pathways known to be important in astrocyte signaling. However, it is still critical to properly validate these tools specifically in astrocytes, especially since they were developed and have been more widely used in neurons. While astrocytic Gq-DREADD activation can increase Ca^2+^ under anesthesia (Durkee et al., 2019; Bonder and McCarthy, 2014), we report for the first time the effect of Gq-DREADD activation on astrocyte Ca^2+^ in awake mice, both for long time periods (2–3 hr) and with several CNO concentrations. Gq-DREADD activation increased Ca^2+^ only for a short time after CNO injection, after which we observe a complete suppression of Ca^2+^ activity for several hours, for all CNO concentrations (Figure 6C–D, Figure 6—figure supplement 1). This unexpected result points to the importance of validating DREADD responses for each in vivo experiment when feasible.

We think that it is most likely that the opposite effects of CNO on Ca^2+^ activity in Gi-DREADD- and Gq-DREADD-expressing astrocytes reveal important differences in the Gi and Gq signaling pathways. However, these results may also indicate that expression levels of Gi- and Gq-DREADD may be more different than expected based on the observed immunostaining. Another possibility is that differential Ca^2+^ responses may be caused by differences in CNO action on Gi- and Gq-DREADD receptors. For example, CNO may be less efficacious on Gi-DREADDs in astrocytes. This possibility could be tested by determining whether higher doses of CNO are sufficient to suppress Ca^2+^ with Gi-DREADD.

### Differential regulation of local and cortex-wide sleep

Two unique slow-waves have been characterized in NREM sleep: delta waves and slow oscillations (Genzel et al., 2014; Kim et al., 2019; Steriade et al., 1993; Steriade and Timofeev, 2003; Siclari et al., 2014; Dang-Vu et al., 2008; Bernardi et al., 2018). Here, we found that astrocytic Gi-DREADD activation increases SWA by preferentially increasing the amplitude of delta waves in V1 (Figure 4). This data supports existing literature suggesting delta waves are generated locally within the cortex by the spreading of DOWN states (Genzel et al., 2014; Siclari and Tononi, 2017; Siclari et al., 2014; Bernardi et al., 2018; Spoormaker et al., 2010; Nir et al., 2011). Since our Gi-DREADD manipulation was restricted within the cortex (Figure 3—figure supplement 1) and DOWN states are thought to be generated through GABAergic inhibition (Chen et al., 2012; Lemieux et al., 2015; Sheroziya and Timofeev, 2014; Zucca et al., 2017), we hypothesize that Gi-GPCR signaling in astrocytes mediates the local synchronization of delta waves via control of inhibition. Additionally, astrocytes may also mediate synchronization of UP states through glutamate (Sanchez-Vives and McCormick, 2000; Poskanzer and Yuste, 2011).

In addition to the increase in delta waves, we observed a smaller, but significant, increase in slow oscillation amplitude that was equal in magnitude to that observed in contralateral FC (Figure 7). This suggests that cortical astrocytes may have influence over more global, cortex-wide neural activity. We explored this further and found that SWA in contralateral FC was modulated around endogenous Ca^2+^ events recorded in V1. Further, we found that coherence between V1 and FC was increased immediately following astrocyte Ca^2+^ events. Interestingly, coherence was increased in the range of 5–10 Hz, which is higher than expected for slow oscillations, but might also indicate a role for astrocytes in the connectivity across cortex during REM or wake. The synchronization across broad areas of cortex may involve astrocytic gap junctions (Szabó et al., 2017; Clasadonte et al., 2017), which could mediate fast recruitment of neurons in synchronous waves. If this is indeed the mechanism, our findings indicate that GPCR activation regulates gap junction coupling in astrocytes, which can be explicitly tested (Murphy-Royal et al., 2020).

Slow oscillations are more global than delta waves. Here, the change in slow oscillation amplitude with CNO administration was similar in V1 and FC (Figure 7J). One explanation for this finding is that activation of V1 astrocytes is sufficient to recruit subcortical circuitry, such as thalamocortical circuits, that can underlie brain-wide synchronous events (Steriade, 2006; Crunelli et al., 2018). This hypothesis is supported by studies showing subcortical ‘bottom-up’ regulation of slow oscillations (Steriade et al., 1993; Siclari et al., 2014; Bernardi et al., 2018) and a role of astrocytes in mediating communication between different brain areas (Kol, 2019; Sardinha et al., 2017), and could be tested by simultaneously recording from thalamus and cortex during astrocyte activation.

Recent work indicates that slow oscillations and delta waves have distinct functions in memory during NREM sleep (Genzel et al., 2014; Kim et al., 2019). Since Gi-driven astrocyte Ca^2+^ preferentially drives changes in delta waves locally, we might expect that astrocytic activity in sleep is more involved in forgetting than memory consolidation. This could be tested by quantifying the effect of Gi-DREADD activation during sleep following a learning paradigm, such as fear conditioning. Further, a light-activated Gi-GPCR (Siuda et al., 2015) in astrocytes would provide temporal control for selective Gi-DREADD activation specifically during NREM sleep following learning, to further explore how astrocytic effects on sleep impact cortical memory functions.

## Materials and methods

### Animals

All procedures were carried out using adult mice (C57Bl/6, P50–100) in accordance with protocols approved by the University of California, San Francisco Institutional Animal Care and Use Committee (IACUC). All animals were housed in a 12:12 light-dark cycle with food and water provided ad libitum. Male and female mice were used for all experiments. IP_3_R2 KO mice (Dr. Katsuhiko Mikoshiba, RIKEN) carry null alleles for *Itpr2*. Following surgery, all animals were singly housed, to protect electrodes, with additional enrichment.

### Surgical procedures

Adult mice (C57Bl/6, P50–100) were administered dexamethasone (5 mg/kg, s.c.) prior to surgery and anesthetized with isoflurane. A custom-made titanium headplate was attached to the skull using C and B Metabond (Parkell), and a 3 mm diameter craniotomy was created over visual cortex. A titanium wire was inserted in V1 lateral to the craniotomy, and a bone screw was inserted in contralateral V1 for reference (all measurements from bregma, −3.5 mm, 1.2 mm lateral). Two twisted titanium wires were inserted in the nuchal muscles for EMG recordings. In a subset of animals, an additional bone screw for EEG was inserted into FC, contralateral to the craniotomy (+2.7 mm, 1.2 mm lateral).

For endogenous Ca^2+^ imaging, two 300 nL injections of *AAV5-GFaABC1D.cyto-GCaMP6f* were made in the brain before placing the cranial window. For Gi-DREADD experiments, two injections of *AAV5-GFaABC1D.cyto-GCaMP6f* (200 nL each, 400 nL total) and *AAV5-GFAP-hM4D(Gi)-mCherry* (100–200 nL each, 200–400 nL total) were co-injected. For Gq-DREADD experiments, two injections of *AAV5-GFaABC1D.cyto-GCaMP6f* (200–300 nL each, 400–600 nL total) and *AAV5-hM3D(Gq)-mCherry* (200–500 nL each, 400–1000 nL total) were injected before placing the cranial window. All injections were 0.2–0.3 mm from the pial surface, −0.5 – −3.5 mm, 1.2–2.5 mm lateral at 30–60 nL/min, followed by a 10 min wait for diffusion. Following viral injection, a glass cranial window for chronic imaging was implanted and secured using C and B metabond (Goldey et al., 2014). Post-operative care included administration of 0.05 mg/kg buprenorphine and 5 mg/kg carpofen. Mice were allowed 10 days to recover, then were habituated to head-fixation on a circular treadmill for 5 days, prior to imaging. For DREADD experiments, mice were habituated for 1–2 days.

### In vivo two-photon imaging and electrophysiology

2P imaging experiments were carried out on a microscope (Bruker Ultima IV) equipped with a Ti:Sa laser (MaiTai, SpectraPhysics). The laser beam was intensity-modulated using a Pockels cell (Conoptics) and scanned with linear galvonometers. Images were acquired with a 16x, 0.8 N.A. Nikon objective via a photomultiplier tube (Hamamatsu) using PrairieView (Bruker) software. For GCaMP imaging, 950 nm excitation and a 515/30 emission filter was used. All recordings started at ZT 2. Mice were head-fixed to a circular treadmill and Ca^2+^ activity was recorded at ~ 1.7 Hz effective frame rate from layer 2/3 of visual cortex with a 512 × 512 pixel resolution at ~ 1 μm/pixel. Locomotion speed was monitored using an optoswitch (Newark Element 14) connected to an Arduino. For LFP and FC-EEG, differential recordings were acquired using the contralateral bone screw as a reference. For EMG, differential recordings were acquired using the two wires implanted in the nuchal muscles. All recordings were amplified (Warner) with a gain of 1K, high-pass filtered at 0.1 Hz, and low-pass filtered at 10 KHz. Electrophysiology and locomotion recordings were acquired simultaneously with 2P imaging at 1 KHz using PrairieView (Bruker) software.

### Image analysis

Astrocyte Ca^2+^ image analysis was performed using Astrocyte Quantitative Analysis (AQuA) software (Wang et al., 2019). Videos were preprocessed by registering images using the ImageJ plugin MOCO (Dubbs et al., 2016). Events were detected using AQuA (in MATLAB) using the in-vivo-GCaMP-cyto preset. Signal detection threshold was adjusted for each video after manually checking for accurate detection, to account for slight differences in noise. AQuA outputs were further analyzed in MATLAB. Event count was quantified using the onset of each event, as detected by AQuA. For ROI analysis, somatic traces were extracted from ROIs hand-drawn using the blow-lasso tool in Fiji. Somatic ROIs were then removed using a mask, and process ROIs were created by applying 10 um^2^ tiles across the field of view.

### Sleep scoring

LFP and EEG recordings were first manually inspected for movement artifacts, which were removed by excluding data exceeding 5 SD from the mean. Similarly, drifting baselines were adjusted by a high-pass filter with a cut-off frequency of 0.3 Hz. All electrophysiology acquired on the same day were pooled together and z-scored. A spectrogram was then calculated using a moving window of 10 s, stepping every 5 s. Locomotory data were used to identify each 5 s bin as *stationary* if no locomotion was detected, or *locomotory* otherwise. The absolute value of z-scored EMG recordings was used to quantify mean EMG amplitude for each 5 s bin.

A bin was identified as NREM sleep if (1) the slow-wave ratio (0.5–4 Hz/8–20 Hz) was > 0.5 SD from the mean, (2) the animal was stationary, and (3) the EMG was < 5 SD from the mean. Similarly, a bin was identified as REM sleep if it had not been quantified as NREM, and (1) theta power (6–10 Hz)>0.25 SD from the mean, (2) the animal was stationary, and (3) the EMG was < 0.4 SD from the mean. All remaining times were characterized as wake. Each behavioral period was identified by finding the start and end of consecutive 5 s bins of the same behavioral state. For all analysis, sleep periods < 10–15 s were excluded, with the exception of the sleep/wake transition analysis (Figure 5), in which sleep periods < 30 s were excluded. Each wake period was further divided into 1 s bins, and characterized as *stationary* if no movement was detected and *locomotory* otherwise. Consecutive 1 s bins of locomotion within each wake period were identified as a *locomotory wake* period and consecutive 1 s bins of no locomotion within each wake period were identified as a *stationary wake* period. Stationary wake periods < 15 s were excluded. We included a ‘buffer’ in which the first 10 s of a stationary wake period was excluded if that stationary wake period immediately followed a locomotory wake period because Ca^2+^ bursts during locomotion often persist for ~ 10 s after locomotion ceases.

### Slow oscillation and delta wave detection

To differentiate slow oscillations and delta waves, we set thresholds for amplitude and peak-to-trough duration (Kim et al., 2019; Siclari et al., 2014; Dang-Vu et al., 2008; Nir et al., 2011; Riedner et al., 2007). First, LFP and EEG was filtered for the slow wave band (0.1–4 Hz) using two filters: a high-pass Butterworth filter (second order, cutoff at 0.1 Hz) and a low-pass Butterworth filter (fourth order, cut-off at 4 Hz). Next, we identified all positive-to-negative zero crossings, preceding peaks, and following troughs that occurred during NREM sleep. Because gamma oscillations are nested in UP states (Steriade et al., 1996; Valderrama et al., 2012; Wolansky et al., 2006; Mena-Segovia et al., 2008), we used high-gamma to verify that the identified peaks were DOWN states and the troughs were UP states: LFP and EEG recordings were bandpass filtered for 80–100 Hz, and high-gamma amplitude was quantified during peaks and troughs. If the mean peak high-gamma was greater than the mean trough high-gamma, we inverted the signal and repeated the analysis.

Slow oscillations were identified as zero crossings with (1) preceding peaks >85th percentile, (2) following troughs <40th percentile, and (3) peak-to-trough duration of 150–500 ms. Delta waves were identified as zero crossings with (1) preceding peaks <85th percentile, (2) following troughs <40th percentile, and (3) peak-to-trough duration > 100 ms.

### In vivo *DREADD activation*

At the start of each experiment (ZT 2), mice were weighed and head-fixed on the treadmill. Leads from the amplifier were connected to the LFP, EEG, and EMG electrodes. A 10 min baseline recording was acquired first, prior to any injection. When this baseline recording was completed, CNO or saline (0.9%) was administered (I.P.). The imaging/recording began immediately after injection. CNO was diluted in saline from a stock of 60 mM each day and a volume was measured for the desired dose (0.1–1.0 mg/kg). An equal volume of saline was injected on control days. The sequence of CNO and saline control days was randomized amongst mice.

### Ex vivo *2P imaging*

For acute slice experiments, neonatal mice (C57Bl/6, P0–4) were anesthetized by crushed ice anesthesia for 3 min and injected with *AAV5-GFaABC1D.cyto-GCaMP6f* at a rate of 2–3 nL/s. Six injection sites (0.5 μm apart in a 2 × 3 grid pattern, at 0.8 μm and 0.15–0 μm below the pial surface) over assumed V1 were chosen. 30 nL/site (360 nL total) was injected with a microsyringe pump (UMP-3, World Precision Instruments). Coronal, acute V1 slices (400 μm thick) from P25–P30 mice were cut with a vibratome (VT 1200, Leica) in ice-cold cutting solution (in mM): 27 NaHCO_3_, 1.5 NaH_2_PO_4_, 222 sucrose, 2.6 KCl, 2 MgSO_4_, 2 CaCl_2_. Slices were incubated in standard continuously aerated (95% O_2_/5% CO_2_) artificial cerebrospinal fluid (ACSF) containing (in mM): 123 NaCl, 26 NaHCO_3_, 1 NaH_2_PO_4_, 10 dextrose, 3 KCl, 2 CaCl_2_, 2 MgSO_4_, heated to 37°C and removed from water bath immediately before introducing slices. Slices were held in ACSF at room temperature until imaging. Experiments were performed in continuously aerated, standard ACSF. 2P imaging was carried out as for in vivo imaging described above. Experiments began with a 10 min incubation in 1 μM TTX, followed by a 2 min baseline video to record spontaneous activity. To record responses to the wake cocktail (20 μM norepinephrine, 5 μM acetylcholine, 5 μM dopamine, 2.5 μM histamine), a 5 min video was acquired in which the cocktail was added to the bath at the start. The frame at which the cocktail entered the imaging chamber was recorded for each experiment. A second video was then acquired, repeating bath-application of the wake cocktail.

### Principal components analysis

To quantify differences in endogenous astrocyte Ca^2+^ events across behavioral periods, PCA was used to reduce the 20 AQuA outputs to 5 PCs, accounting for 73% of the variance in the original event features. The 5 PC scores for each astrocyte event in each behavioral state across all mice were used to construct empirical cumulative distribution functions (CDFs) for each PC in each behavioral state. The CDFs for stationary wake and sleep were compared for each PC using a two-sample Kolmogorov-Smirnov (K-S) test. In Gi-DREADD experiments, separate PCA was performed to reduce the 20 output features, as above, to 5 PCs accounting for 69% of the variance. For each PC, and for each mouse, four empirical CDFs were constructed from the PC scores of recorded events, corresponding to each combination of saline or CNO as intervention, and NREM sleep or wake as behavioral state. Within each behavioral state, the K-S distance was computed between the corresponding saline and CNO distributions, to estimate the effect of CNO administration on the given PC’s score distribution during that behavioral period. To assess differences between these effects in different behavioral states for a given PC, the K-S distances between the distributions in saline and CNO trials across all mice were compared between NREM sleep and wake using a Wilcoxon signed-rank test.

### Quantifying coherence

To quantify functional connectivity between V1 and FC, we computed spectral coherence between the two signals in saline-administration sessions. Coherence spectra were calculated in a 1.5 s window following the onset of astrocyte events using Welch’s averaged periodogram method, utilizing a Hann window and a fast Fourier transform size of 1024 samples, as implemented in SciPy. Event-aligned coherence spectra were compared against coherence spectra aligned to randomly chosen time-points. Random time-points were chosen uniformly between the start of each dataset and 1.5 s (the duration of the coherence analysis window) from the end, in equal numbers to the original astrocyte events for each dataset. To compare between astrocyte- and random-aligned coherence, each mouse’s median event-aligned and random-aligned coherence spectra were calculated; a paired two-tailed *t*-test was then performed at each frequency between the event-aligned median values and corresponding random-aligned median values for all mice. To control the familywise error rate across compared frequencies, the Bonferroni correction was applied to the resulting *p*-values.

This analysis was extended to quantify coherence changes at sleep-to-wake transitions. Coherence spectra were computed as above in a 1.5 s window following astrocyte events during NREM sleep, and following uniformly randomly selected events that coincided with NREM sleep. These spectra were separated into bins based on proximity of the corresponding event onset (or random alignment point) to the next sleep-to-wake transition, with each bin encompassing 2 s relative to the transition; the window most proximal to the transition was truncated to 0.5 s to avoid overlap of the 1.5 s window for coherence computation with the subsequent wake period. Coherence values within 0–15 Hz were averaged in each time bin; averaged coherence values were then compared between the astrocyte- and random-aligned cases in each bin using an unpaired two-tailed *t*-test. To control the familywise error rate across compared time bins, the Bonferroni correction was applied to the resulting p-values.

### Immunohistochemistry

After physiology experiments were complete, mice were intracardially perfused with 4% PFA. Brains were collected, immersed in 4% PFA overnight at 4°C and switched to 30% sucrose for 2 days before being frozen on dry ice and stored at −80°C. Brains were sliced coronally (40 μm thick) on a cryostat. Slices were stored in cryoprotectant at −20°C until staining. 17–24 slices/mouse were chosen to span from +2.8 to −4.24 mm from bregma; each slice was 280 μm from the proximate slice. Slices were washed with PBS, 5 min x 3, then with 0.1% PBS-TX for 30 min. Slices were next washed with 10% NGS (Invitrogen) for 1 hr, followed by an overnight incubation of 2% NGS, rat α-mCherry (1:1000, ThermoFischer), rabbit α-NeuN (1:1000, EMD Millipore), and chicken α-GFP (1:3000, Aves Lab) in 4°C. Slices were next rinsed with 1x PBS x three before incubating for 2 hr at room temperature with goat α-rat Alexa Fluor 555 (1:1000), goat α-rabbit 405 (1:1000), and goat α-chicken Alexa Fluor 488 (1:1000). Slices were washed again with PBS 3x for 5 min before slide-mounting and coverslipping using Fluoromount.

Whole coronal slice images were taken using an AxioImager Z2 upright epiflorescent microscope (Zeiss). 5x images were acquired, and z-stacks were stitched together with Zen Software. Images were segmented using WEKA (Arganda-Carreras et al., 2017): A classifier was trained to segment 5x images into three classes: (1) pixels containing fluorescence, (2) pixels containing non-fluorescent brain tissue, and (3) pixels containing background. The classifier was then applied to the full dataset, and images were checked manually for accurate segmentation. Each segmented image was manually divided in Fiji to isolate each hemisphere. Quantification of viral spread was calculated in MATLAB by normalizing the number of fluorescent pixels to the number non-fluorescent pixels within the tissue for each hemisphere.

To analyze colocalization of mCherry and NeuN at single-cell resolution, 63x images were taken on a spinning disk confocal (Zeiss). Slides were oil-immersed and two slices/animal (−3.8 and −2.3 from bregma) were imaged. In these slices, eight images were taken at random, spanning the total area in which virus was expressed. Colocalization of mCherry and NeuN was performed using Fiji.

### Quantification and statistical analysis

All statistical tests used, definition of center and dispersion measurements, and exact n values can be found for each figure in the corresponding figure legend. Additional information regarding statistical tests described in the relevant sections. For all figures, significance levels defined as the following: *: p<0.05, **: p<0.005, ***: p<0.0005.

## Data Availability

All datasets are available on Dryad. Analysis code is available on GitHub. The following dataset was generated: KiraP TrishaV 2020Cortical astrocytes independently regulate sleep depth and duration via separate GPCR pathwaysDryad Digital Repository10.7272/Q6C53J39PMC796892733729913 The following previously published datasets were used: ZhangY ChenK SloanSA BennettML ScholzeAR O'KeeffeS PhatnaniHP GuarnieriP CanedaC RuderischN DengS LiddelowSA ZhangC DanemanR ManiatisT BarresBA WuJQ 2014Brain RNA-SeqBrain RNA-Seqbrainrnaseq10.1523/JNEUROSCI.1860-14.2014PMC415260225186741 AdultAstrocyte RNA-Seq Explorer ChaiH Diaz-CastroB ShigetomiE MonteE OcteauJC YuX CohnW RajendranPS VondriskaTM WhiteleggeJP CoppolaG KhakhBS 2017Adult Astrocyte RNA-Seq Explorern/aastrocyternaseq10.1016/j.neuron.2017.06.029PMC581131228712653

## Additional files

Transparent reporting form
