# Supplementary material for: Cortical astrocytes independently regulate sleep depth and duration via separate GPCR pathways
Source: eLife. 2021 Mar 17;10:e63329. doi: 10.7554/eLife.63329 (PMC7968927; doi:10.7554/eLife.63329)
Supplement: Peer review history

# Decision letter

**DOI:** 10.7554/eLife.63329.sa1

**Affiliations:** Boston Children's Hospital United States

In the interests of transparency, eLife publishes the most substantive revision requests and the accompanying author responses.

**Acceptance summary:**

This is an exciting paper demonstrating roles for cortical astrocytes in sleep. Activity in networks of neurons throughout the brain is different when an animal is asleep compared to when it is awake, and it has long been speculated that astrocytes, non-neuronal cells in the brain, may be partly responsible for regulating neuronal network activity during sleep. However, solid evidence has been largely lacking until recently. This paper plays an important in elucidating the connection between astrocytes and sleep, and suggests that astrocytes play a homeostatic role in regulating neurons during sleep.

**Decision letter after peer review:**

Thank you for submitting your article "Cortical astrocytes independently regulate sleep depth and duration via separate GPCR pathways" for consideration by *eLife*. Your article has been reviewed by two peer reviewers, and the evaluation has been overseen by a Reviewing Editor and Laura Colgin as the Senior Editor. The reviewers have opted to remain anonymous.

The reviewers have discussed the reviews with one another and the Reviewing Editor has drafted this decision to help you prepare a revised submission.

We would like to draw your attention to changes in our revision policy that we have made in response to COVID-19 (https://elifesciences.org/articles/57162). Specifically, when editors judge that a submitted work as a whole belongs in *eLife* but that some conclusions require a modest amount of additional new data, as they do with your paper, we are asking that the manuscript be revised to either limit claims to those supported by data in hand, or to explicitly state that the relevant conclusions require additional supporting data.

Our expectation is that the authors will eventually carry out the additional experiments and report on how they affect the relevant conclusions either in a preprint on bioRxiv or medRxiv, or if appropriate, as a Research Advance in *eLife*, either of which would be linked to the original paper.

Summary

This work builds on several recent publications implicating astrocyte control of sleep but represents an several new findings that provide new insight toward elucidating the mechanisms underlying the reported observations. The paper presents high quality data, rigorous experiments and analyses. Overall the paper is well written; however, there are several (relatively straightforward) points to address prior to publication as summarized below from both reviewers.

Revisions for this paper:

– Please revise to increase clarity in text and figures as suggested.

– The temporal relationships between astrocyte signals and SWA changes is complex, with SWA decreasing before astrocyte calcium events and increasing after. This is interpreted as astrocytes having a homeostatic effect on SWA. Causality is therefore somewhat hard to interpret, in part because the homeostatic effect may involve more than just astrocytes but also neurons or other cell types. Including imaging data of cortical astrocytes together with nearby cortical neurons could help clarify the temporal relationship between these cells. If this data is not available, please address this possibility.

– Along similar lines, the authors demonstrate sufficiency, but do not conclusively demonstrate that sleep is regulated through these pathways because of the difficulty in knocking them out. Please temper claims and address alternative interpretations in the Discussion.

– Is CNO is equally efficacious at Gi and Gq DREADDs for downstream signaling in astrocytes? Please comment or determine if the Gi and Gq DREADDs are expressed to the same level in astrocytes (see points 3,4 reviewer #1 and related comments by reviewer 2).

– In this study SWA, measured through LFP, is used as the primary measure of sleep; however SWA can also be measured from EEG, a more common way to define sleep states. Can data be included that quantifies the degree to which these are correlated? Other indicators of sleep include arousal threshold could also help show relationships between their LFP-measure of SWA and such other measures? Please include or address potential caveats in paper if data cannot be included.

*Reviewer #1:*

The paper by Poskanzer and colleagues presents a detailed description of astrocyte calcium dynamics during natural sleep and then explores the consequences on slow wave activity (SWA) of stimulating astrocyte Gi and Gq DREADDs. Overall, this study builds on several years' worth of work by many labs showing or at least suggesting potentially important roles of astrocytes in sleep. As such, the topic and the data are not completely novel, but to their credit the authors have performed in depth analyses that go far deeper than previous studies. The depth of the analyses and the rigor of the approach outweigh any concerns that may linger regarding past work and push this study into a category of paper that is much needed and valued in the field. Seems highly appropriate for *eLife*.

No major concerns.

*Reviewer #2:*

This is an exciting paper showing relationships between sleep and Gi- and Gq-coupled GPCR signaling pathways in astrocytes. The paper demonstrates correlations between astrocyte calcium signals and sleep signatures such as slow-wave activity (SWA) measured with LFP in cortex. Astrocytes show activity changes near SWA changes such as at sleep-wake transitions. These are investigated using Gi- and Gq-DREADDs and it is found that astrocytes have a causal effect on SWA and the frequency of sleep-wake transitions. These results provide evidence for a role for astrocytes in controlling sleep-related SWA, with separate effects on sleep depth and duration depending on which GPCR pathway is perturbed.

This work is related to other recent publications implicating astrocyte control of sleep and contains a multitude of new findings. These are very interesting and elucidating while also pointing toward directions of research needed for determining the molecular and mechanisms underlying the reported observations.

1) The conclusions are somewhat indirect when it comes to causal relationships and mechanisms. The temporal relationships between astrocyte signals and SWA changes is complex, with SWA decreasing before astrocyte calcium events and increasing after. This is interpreted as astrocytes having a homeostatic effect on SWA. Causality is therefore somewhat hard to interpret, in part because the homeostatic effect may involve more than just astrocytes but also neurons, including neurons whose activity is less visible in LFP measurements of SWA. Although this may be outside the scope of this study, if the authors have imaging data available of cortical astrocytes together with nearby cortical neurons (that is, a more direct measure of nearby neuron activity than LFP), this might clarify the temporal relationship between these cells.

2) The paper demonstrates sufficiency, astrocytes can change cortical SWA and sleep duration, but does not conclusively demonstrate that sleep is regulated through these pathways in natural behavior because of the difficulty in knocking them out. Can the text (perhaps the title) be adjusted to reflect this?

3) The authors show that there is inverse relationship between SWA amplitude and astrocytic calcium events in Figure 1H, with higher calcium activities associated with lower SWA. Gi-DREADD activation by CNO can increase astrocytic calcium events, and also increase SWA. Can the authors discuss this changed relationship in more depth? In addition, Gq-DREADD can change sleep time and sleep-wake transition, but not change SWA. Do these results indicate that astrocytic calcium may be not directly affect sleep duration or depth, but some other signal downstream of the GPCR?

4) In the Gi-DREADD experiments, the IP3R2-/- mouse is tested and found to lead to a smaller effect of CNO on SWA. Why was the IP3R2-/- not included in the Gq-DREADD experiments? Was that based on the assumption that there would be no difference, since Ca events disappeared in the Gq experiments?

5) It is unclear what the true effect of the Gq-DREADDs is on astrocyte calcium because activity is only represented by event rate (extracted by AQuA) and not as Ca concentration. Does event rate go down because Ca concentration drops to low levels and shows no dynamics, or because Ca concentration saturates and shows no dynamics due to a ceiling effect (as in the wake cocktail experiments)? This could be measured as absolute fluorescence of GCaMP. Interpretation of the results could change depending on which one of these is true. For example, saturated calcium could indicate an “unphysiological” state of astrocytes that can have consequences on the network that may not occur during natural sleep/wake.

6) The conclusion that "Gq-induced Ca^2+^ is necessary for sleep-wake transitions" (text and Figure 6) seems somewhat overstated. The Gq-DREADDs are a useful way to perturb calcium dynamics and this leads to changes in sleep-wake transitions, but it was not shown that without Gq sleep-wake transitions disappear. Also the effect demonstrated (Figure 6F for example) is partial. It seems appropriate to rephrase this statement.

7) SWA, measured through LFP, is used as the primary measure of sleep. SWA can also be measured from EEG, and this appears the more common way to define sleep states. Can data be included that quantifies the degree to which these are correlated? Is cortical LFP-measured SWA one hundred percent predictive of SWA derived from EEG? Other indicators of sleep include arousal threshold. Do the authors have data showing relationships between their LFP-measure of SWA and such other measures?

8) Related to this, how are changes in LFP-measured SWA in local cortical areas related to EEG-measured SWA during perturbation experiments? This is partially addressed in Figure 7 by measuring SWA with LFP in another cortical area, but it would be interesting to compare this to a global EEG measure.

9) Is there any possibility that manipulation of astrocytes may directly influence LFP, for example through secretion of ions? It seems unlikely, but not impossible.

# Reply: Author response

**DOI:** 10.7554/eLife.63329.sa2

Revisions for this paper:

– Please revise to increase clarity in text and figures as suggested.

As suggested by the reviewers, we have edited the manuscript for clarity throughout the paper, focusing most heavily on the sections that they commented on the most, but making changes in all sections to soften the tone and clarify the analyses and conclusions.

– The temporal relationships between astrocyte signals and SWA changes is complex, with SWA decreasing before astrocyte calcium events and increasing after. This is interpreted as astrocytes having a homeostatic effect on SWA. Causality is therefore somewhat hard to interpret, in part because the homeostatic effect may involve more than just astrocytes but also neurons or other cell types. Including imaging data of cortical astrocytes together with nearby cortical neurons could help clarify the temporal relationship between these cells. If this data is not available, please address this possibility.

We agree that we cannot conclude causality from the endogenous dataset alone. We have adjusted the language in the text to emphasize that our interpretation regarding the role of astrocytes in SWA homeostasis is speculative and that we cannot rule out the role of other cell types in this process, as we indeed believe (see Results). While we believe that dual-color imaging of astrocytes and neighboring neurons would certainly reveal more about the temporal relationship between these cell populations, it is outside of the scope of this project. However, we are in the process of preparing for such experiments in the lab for the future.

– Along similar lines, the authors demonstrate sufficiency, but do not conclusively demonstrate that sleep is regulated through these pathways because of the difficulty in knocking them out. Please temper claims and address alternative interpretations in the Discussion.

We have added several sentences to the Discussion to explicitly discuss the use of astrocyte-specific knock-outs in demonstrating necessity, which the reviewer correctly points out were outside of the scope of this study. As such, we also adjusted text throughout to temper these claims. To address alternative interpretations, we also added text to clarify that without evidence of necessity, we do not know whether other signaling cascades or cell types are needed to regulate SWA.

– Is CNO is equally efficacious at Gi and Gq DREADDs for downstream signaling in astrocytes? Please comment or determine if the Gi and Gq DREADDs are expressed to the same level in astrocytes (see points 3,4 reviewer #1 and related comments by reviewer 2).

We do not know whether CNO is equally efficacious for the Gi and Gq DREADDs, but agree that this would be important to fully understand what is causing the differential Ca^2+^ responses to DREADD activation. We have added this possibility to the text (see Discussion), specifically emphasizing that we cannot conclude what is the cause of this change and describing alternative interpretations if we performed the experiments with different CNO doses as described by reviewer #1.

We agree that it is interesting to compare the results of Gq-DREADD activation on event rate with that of fluorescence levels. As such, we have added a panel (Figure 6—figure supplement 1I) in which we used ROIs to analyze change in fluorescence after CNO administration in somas and processes. We see that Ca^2+^ concentration in both somas and processes remain elevated after CNO administration. This could, as the author suggests, indicate Ca^2+^ is clamped at a saturating level. We believe this does not change our interpretation. As is the case with all DREADD manipulations, we are altering astrocyte activity in a “non-physiological” way. Regardless of the exact mechanism, the perturbation that causes a decrease in event rate and a change in sleep—in combination with our endogenous, “physiological” activity (see Figure 4)—informs our understanding of the potential role of astrocytes in this process.

Lastly, while we agree quantifying DREADD protein levels with western blots would be informative, it was currently beyond the scope of this study. We did, however, indirectly compare expression levels between Gi- and Gq-DREADDs by analyzing the immunohistochemistry data we have collected (see Author response image 1). Specifically, we analyzed the pixel intensity values of mCherry fluorescence from samples collected from all mice (Gi: n = 10 mice; Gq: n = 12 mice). We analyzed images taken with both 5x and 63x magnification, and found no significant difference between fluorescence using this approach (ranksum test, p > 0.05). As a note, we did observe that the virus for Gi-DREADDs expressed more strongly than Gq-DREADDs during pilot experiments and so we injected a lower volume of the Gi-DREADD virus (*AAV5-GFAP-hM4D(Gi)-mCherry,* 200-400nl) than the Gq-DREADD virus (*AAV5-GFAP-hM3D(Gq)-mCherry* 400-600nl, see Materials and methods).

**Author response image 1.**

– In this study SWA, measured through LFP, is used as the primary measure of sleep; however SWA can also be measured from EEG, a more common way to define sleep states. Can data be included that quantifies the degree to which these are correlated? Other indicators of sleep include arousal threshold could also help show relationships between their LFP-measure of SWA and such other measures? Please include or address potential caveats in paper if data cannot be included.

While it is true that LFP and EEG will produce different neural recordings, we expect that patterns of SWA and sleep scoring will be very similar between LFP and EEG recordings. In fact, the use of LFP to define sleep states and quantify SWA is quite common in rodents (see for example, Watson et al., 2016, Hengen et al., 2016, Kim et al., 2019). The main difference between LFP and EEG is the spatial scale of the recording, with LFP being a more local signal. However, it has been shown that the spatial scale of the recording is inversely related to the frequency, and thus we would expect that the spatial scale of the LFP is more similar to EEG for the low-frequencies we used to define sleep states and measure SWA and indeed it has been shown that these recordings are largely similar (for further discussion on this, see review Buzsaki, Anastassioiu and Koch 2012). In our data, we found that, using a 5 second window, the average power correlation for SWA (0.5-4 Hz) in LFP (V1) and EEG (FC) was relatively high (See Author response image 2. Saline controls: n = 13 mice, 26 hours, r = 0.67, p < 0.0005; Gi-DREADDs + CNO: n = 19 mice, 38 hours, r = 0.69, p <0.0005). We also found that the sleep state recorded in the V1 LFP and FC EEG agreed in 80% of our timebins. Thus even in two different sites, we find that these two signals are largely similar. The remaining differences we see in sleep state and SWA may reflect local sleep and the heterogeneity of SWA that has been reported, and which we were specifically interested in capturing for the purposes of the present study.

**Author response image 2.**

Reviewer #2:

This is an exciting paper showing relationships between sleep and Gi- and Gq-coupled GPCR signaling pathways in astrocytes. The paper demonstrates correlations between astrocyte calcium signals and sleep signatures such as slow-wave activity (SWA) measured with LFP in cortex. Astrocytes show activity changes near SWA changes such as at sleep-wake transitions. These are investigated using Gi- and Gq-DREADDs and it is found that astrocytes have a causal effect on SWA and the frequency of sleep-wake transitions. These results provide evidence for a role for astrocytes in controlling sleep-related SWA, with separate effects on sleep depth and duration depending on which GPCR pathway is perturbed.

This work is related to other recent publications implicating astrocyte control of sleep and contains a multitude of new findings. These are very interesting and elucidating while also pointing toward directions of research needed for determining the molecular and mechanisms underlying the reported observations.

1) The conclusions are somewhat indirect when it comes to causal relationships and mechanisms. The temporal relationships between astrocyte signals and SWA changes is complex, with SWA decreasing before astrocyte calcium events and increasing after. This is interpreted as astrocytes having a homeostatic effect on SWA. Causality is therefore somewhat hard to interpret, in part because the homeostatic effect may involve more than just astrocytes but also neurons, including neurons whose activity is less visible in LFP measurements of SWA. Although this may be outside the scope of this study, if the authors have imaging data available of cortical astrocytes together with nearby cortical neurons (that is, a more direct measure of nearby neuron activity than LFP), this might clarify the temporal relationship between these cells.

See above, revision # 2

2) The paper demonstrates sufficiency, astrocytes can change cortical SWA and sleep duration, but does not conclusively demonstrate that sleep is regulated through these pathways in natural behavior because of the difficulty in knocking them out. Can the text (perhaps the title) be adjusted to reflect this?

See above, revision # 3

3) The authors show that there is inverse relationship between SWA amplitude and astrocytic calcium events in Figure 1H, with higher calcium activities associated with lower SWA. Gi-DREADD activation by CNO can increase astrocytic calcium events, and also increase SWA. Can the authors discuss this changed relationship in more depth? In addition, Gq-DREADD can change sleep time and sleep-wake transition, but not change SWA. Do these results indicate that astrocytic calcium may be not directly affect sleep duration or depth, but some other signal downstream of the GPCR?

We believe that this relationship fits with our hypothesis that astrocytes are part of a homeostatic process to regulate SWA. In response to decreases in SWA, astrocyte Ca^2+^ events are triggered, which we see with the inverse correlations in Figure 1. We believe that these Ca^2+^ events can then cause a subsequent increase in SWA. In Figure 3, we are artificially increasing Ca^2+^ events above levels seen endogenously, and as a result we are driving an increase in SWA above levels seen in controls. We hope that this explanation, although still speculative, better explains the relationships we observe in Figure 1 and 3. We have added text to the Results section after the explanation of Figure 3 describing this for the reader.

We agree that the functional dissociation seen between manipulation of Gi- and Gq-induced Ca^2+^ suggests that Ca^2+^ is likely not the only signaling molecule driving these changes, and that the many other signaling molecules that are downstream of the GPCRs may play an important role. We have added this to our discussion and the Results section for Figure 6.

4) In the Gi-DREADD experiments, the IP3R2-/- mouse is tested and found to lead to a smaller effect of CNO on SWA. Why was the IP3R2-/- not included in the Gq-DREADD experiments? Was that based on the assumption that there would be no difference, since Ca events disappeared in the Gq experiments?

As the reviewer correctly states, we decided that given Gq-DREADD activation blocked Ca^2+^ events, we would not see a further depletion with the IP3R2-/- mice and interpreting the results on sleep behavior would be difficult.

5) It is unclear what the true effect of the Gq-DREADDs is on astrocyte calcium because activity is only represented by event rate (extracted by AQuA) and not as Ca concentration. Does event rate go down because Ca concentration drops to low levels and shows no dynamics, or because Ca concentration saturates and shows no dynamics due to a ceiling effect (as in the wake cocktail experiments)? This could be measured as absolute fluorescence of GCaMP. Interpretation of the results could change depending on which one of these is true. For example, saturated calcium could indicate an “unphysiological” state of astrocytes that can have consequences on the network that may not occur during natural sleep/wake.

See above, revision # 4

6) The conclusion that "Gq-induced Ca^2+^ is necessary for sleep-wake transitions" (text and Figure 6) seems somewhat overstated. The Gq-DREADDs are a useful way to perturb calcium dynamics and this leads to changes in sleep-wake transitions, but it was not shown that without Gq sleep-wake transitions disappear. Also the effect demonstrated (Figure 6F for example) is partial. It seems appropriate to rephrase this statement.

We agree with the reviewer that because we did not see transitions completely disappear with our Gq manipulation, we cannot necessarily conclude that Gq-induced Ca^2+^ is necessary for sleep-wake transitions. We have changed the use of the world “necessary” to “important” to reflect this.

7) SWA, measured through LFP, is used as the primary measure of sleep. SWA can also be measured from EEG, and this appears the more common way to define sleep states. Can data be included that quantifies the degree to which these are correlated? Is cortical LFP-measured SWA one hundred percent predictive of SWA derived from EEG? Other indicators of sleep include arousal threshold. Do the authors have data showing relationships between their LFP-measure of SWA and such other measures?

See above, revision # 5

8) Related to this, how are changes in LFP-measured SWA in local cortical areas related to EEG-measured SWA during perturbation experiments? This is partially addressed in Figure 7 by measuring SWA with LFP in another cortical area, but it would be interesting to compare this to a global EEG measure.

See above, revision # 5

9) Is there any possibility that manipulation of astrocytes may directly influence LFP, for example through secretion of ions? It seems unlikely, but not impossible.

We agree that it is possible astrocytic control of ions may underlie the observed changes in LFP. Astrocytes are well known to regulate extracellular ion concentrations, which has been implicated in regulating sleep (for example see Ding et al., 2016) and slow-wave activity specifically (for example see Amzica and Steriade, 2000).
